# Development and external validation of a risk prediction model for falls in patients with an indication for antihypertensive treatment: retrospective cohort study

**DOI:** 10.1136/bmj-2022-070918

**Published:** 2022-11-08

**Authors:** Lucinda Archer, Constantinos Koshiaris, Sarah Lay-Flurrie, Kym I E Snell, Richard D Riley, Richard Stevens, Amitava Banerjee, Juliet A Usher-Smith, Andrew Clegg, Rupert A Payne, F D Richard Hobbs, Richard J McManus, James P Sheppard, STRAtifying Treatments In the multi-morbid Frail elderlY (STRATIFY) investigators, John Gladman, Simon Griffin, Margaret Ogden

**Affiliations:** 1 Centre for Prognosis Research, School of Medicine, Keele University, Keele, UK; 2 Nuffield Department of Primary Care Health Sciences, University of Oxford, Oxford, OX2 6GG, UK; 3 Institute of Health Informatics, University College London, London, UK; 4 Primary Care Unit, Department of Public Health and Primary Care, University of Cambridge, UK; 5 Academic Unit for Ageing and Stroke Research, Bradford Institute for Health Research, University of Leeds, UK; 6 Centre for Academic Primary Care, Population Health Sciences, University of Bristol, Bristol, UK

## Abstract

**Objective:**

To develop and externally validate the STRAtifying Treatments In the multi-morbid Frail elderlY (STRATIFY)-Falls clinical prediction model to identify the risk of hospital admission or death from a fall in patients with an indication for antihypertensive treatment.

**Design:**

Retrospective cohort study.

**Setting:**

Primary care data from electronic health records contained within the UK Clinical Practice Research Datalink (CPRD).

**Participants:**

Patients aged 40 years or older with at least one blood pressure measurement between 130 mm Hg and 179 mm Hg.

**Main outcome measure:**

First serious fall, defined as hospital admission or death with a primary diagnosis of a fall within 10 years of the index date (12 months after cohort entry). Model development was conducted using a Fine-Gray approach in data from CPRD GOLD, accounting for the competing risk of death from other causes, with subsequent recalibration at five and 10 years using pseudo values. External validation was conducted using data from CPRD Aurum, with performance assessed through calibration curves and the observed to expected ratio, C statistic, and D statistic, pooled across general practices, and clinical utility using decision curve analysis at thresholds around 10%.

**Results:**

Analysis included 1 772 600 patients (experiencing 62 691 serious falls) from CPRD GOLD used in model development, and 3 805 366 (experiencing 206 956 serious falls) from CPRD Aurum in the external validation. The final model consisted of 24 predictors, including age, sex, ethnicity, alcohol consumption, living in an area of high social deprivation, a history of falls, multiple sclerosis, and prescriptions of antihypertensives, antidepressants, hypnotics, and anxiolytics. Upon external validation, the recalibrated model showed good discrimination, with pooled C statistics of 0.843 (95% confidence interval 0.841 to 0.844) and 0.833 (0.831 to 0.835) at five and 10 years, respectively. Original model calibration was poor on visual inspection and although this was improved with recalibration, under-prediction of risk remained (observed to expected ratio at 10 years 1.839, 95% confidence interval 1.811 to 1.865). Nevertheless, decision curve analysis suggests potential clinical utility, with net benefit larger than other strategies.

**Conclusions:**

This prediction model uses commonly recorded clinical characteristics and distinguishes well between patients at high and low risk of falls in the next 1-10 years. Although miscalibration was evident on external validation, the model still had potential clinical utility around risk thresholds of 10% and so could be useful in routine clinical practice to help identify those at high risk of falls who might benefit from closer monitoring or early intervention to prevent future falls. Further studies are needed to explore the appropriate thresholds that maximise the model’s clinical utility and cost effectiveness.

## Introduction

The proportion of older adults in the population is rising,^1^ and with age the risk of falls increases,^2^ ^3^ which can result in serious injury and long term disability.^4^ In England, falls are associated with about 235 000 emergency hospital admissions in the over 65s and cost the National Health Service more than £2.3bn ($2.6bn; €2.6bn) every year.^5^ ^6^ ^7^

Many risk factors for falls exist, primarily related to comorbidities and frailty.^2^ ^3^ ^8^ ^9^ ^10^ A key modifiable risk factor is prescribed drugs, including those that lower blood pressure.^11^ ^12^ ^13^ Although antihypertensives are effective at reducing the risk of cardiovascular disease, typically many patients require treatment over several years to prevent a small number of events.^14^ Data from randomised controlled trials show that antihypertensives are associated with an increased risk of hypotension and syncope, which may lead to falls.^15^ Observational studies examining patients with frailty and multimorbidity suggest a direct association between antihypertensive treatment and falls.^11^ ^16^ ^17^

In patients who are prescribed antihypertensives or other drugs that substantially increase their risk of falls, doctors might want to consider altering or withdrawing treatment (ie, deprescribing),^18^ along with other interventions to reduce the risk of falls (eg, advice on lower alcohol consumption, falls prevention clinics, exercises).^7^ Identifying people at high risk of falls is, however, challenging. A 2021 systematic review of falls prediction models for use in the community identified a total of 72 models.^10^ Most of these studies were deemed at high risk of bias, and only three of the models were externally validated. These three validated models showed moderate discriminative ability, with an area under the curve of between 0.62 and 0.69. Calibration based on internal validation was only reported in seven of the studies, and it was typically moderate to poor.^10^ A further primary analysis aiming to predict falls in a general practice population showed good apparent discrimination for the model used (with an area under the curve of 0.87), but calibration performance was not assessed and no external validation was performed.^19^

To inform clinical decision making in primary care, both patients and doctors require better prediction models to accurately identify those at high risk of serious falls (defined as any fall resulting in hospital admission or death), from the population of older adults who might be considered for antihypertensive treatment. This population includes patients with a recent high blood pressure reading, including those with a new diagnosis of hypertension, as well as those in whom intensification of treatment is being considered. We used routinely collected data from electronic health records to develop and externally validate a clinical prediction model to estimate such individuals’ risk of experiencing a fall resulting in hospital admission or death within 10 years. This study is part of a broader research programme investigating the association between blood pressure lowering drugs and side effects: STRAtifying Treatments In the multi-morbid Frail elderlY (STRATIFY): Antihypertensives.

## Methods

A retrospective observational cohort study was used to develop a prediction model for serious falls (the STRATIFY-Falls model), using data from Clinical Practice Research Datalink (CPRD) GOLD, which contains information from general practices using Vision electronic health record software (Cegedim Healthcare Solutions, London, UK). The model was externally validated using a second retrospective observational cohort comprising data from CPRD Aurum, containing data from general practices using recording software from Egton Medical Information Systems (EMIS, Leeds, UK). These data were linked to Office for National Statistics mortality data, Hospital Episode Statistics, and index of multiple deprivation data. The CPRD independent scientific advisory committee approved the protocol for this study (protocol No 19_042, see Appendix 6 in the supplementary material).

### Population

Patients were eligible if they were registered at a linked general practice in England, contributing to CPRD between 1 January1998 and 31 December 2018. At the time of analysis, CPRD GOLD (development cohort) contained 4.4 million active patients from 674 general practices, whereas CPRD Aurum (validation cohort) contained seven million active patients from 738 practices. Both datasets have previously been shown to be representative of the patient population in England for age, ethnicity, and deprivation status.^20^ ^21^ To avoid duplication of patients, when practices had switched from one recording system to the other during the study timeframe, we excluded practices from CPRD Aurum (validation cohort) that were also present in the CPRD GOLD (development) dataset.

Patients were considered eligible if they were aged 40 years or older (no upper age limit applied), registered to a CPRD “up-to-standard” practice (CPRD GOLD only), and had records available during the study period. Patients entered the cohorts at the time at which they became potentially eligible for antihypertensive treatment (ie, at the time of their first systolic blood pressure reading ≥130 mm Hg) after the study start date, and they were followed for up to 10 years. This blood pressure threshold was chosen to account for varying treatment initiation thresholds specified in different international hypertension guidelines.^6^ Patients with any systolic blood pressure reading >180 mm Hg were excluded from the cohort, as antihypertensive treatment would be indicated for these patients regardless of the risk of adverse events, unless clearly contraindicated for other reasons. All patient characteristics and model predictors were determined at the index date, defined as 12 months after cohort entry. The same eligibility criteria and characteristic determination methods were applied to both the development cohort and the validation cohort.

### Outcomes

The primary outcome was any hospital admission or death associated with a primary diagnosis of a fall within 10 years of the index date, the same time horizon as used for cardiovascular prediction models.^22^ Falls were based on ICD-10 (international classification of diseases, 10th revision) codes documented in Hospital Episodes Statistics and ONS mortality data (applicable ICD-10 codes shown in supplementary table S4.1). Prespecified secondary outcomes were falls (defined in the same way) within one and five years of the index date. This outcome definition was consistent across both the development cohort and the validation cohort.

### Model predictors

We identified clinically relevant predictors of falls from the literature and through expert clinical opinion.^2^ ^7^ ^8^ ^9^ ^23^ These included 30 predictors (44 predictor variables), covering patient demographics (age, sex, ethnicity, area based socioeconomic deprivation (index of multiple deprivation), body mass index (BMI), systolic and diastolic blood pressure), clinical characteristics (total cholesterol level, smoking status, alcohol intake), comorbidities (previous falls, memory problems, mobility issues, history of stroke, multiple sclerosis, activity limitation, syncope, cataract), and prescribed drugs (antihypertensives, opioids, hypnotics or benzodiazepines, antidepressants, anticholinergics) (see table S4.2 in the supplementary material). A recent literature review of falls clinical prediction tools by the National Institute for Health and Care Excellence identified the need for frailty to be considered as a predictor in models for use in the community.^24^ We therefore also calculated a validated electronic frailty index using the 36 comorbidities and conditions specified, including this index as a single covariate.^25^ Covariates were defined by any occurrence of relevant Read or SNOMED codes at any time point before the index date, with the exception of antihypertensives, which were defined as any prescription in the 12 months before the index date.

To ensure consistency with commonly used risk calculators,^26^ ^27^ our prediction models do not account for changes in prescriptions of drug type or amount over time, and as such give an estimation of falls risk assuming treatment assignment policy in any application setting is similar to that in the development data.^28^

### Sample size

The prespecified sample size calculation for model development was 2194 participants (15 358 person years), assuming a maximum of 40 predictors would be included in the final model (see extended methods in the supplementary material).^29^ For the external validation, the estimated sample size required was 12 000 patients (with at least 708 experiencing falls), sufficient to target a 95% confidence interval of width 0.2 around the estimate of the calibration slope (see extended methods in the supplementary material).^30^ The actual sample sizes in both the development cohort and the validation cohort far exceeded these estimates.

### Statistical analysis

We calculated descriptive statistics for baseline characteristics in the model development and external validation cohorts separately.

### Missing data

Multiple imputation with chained equations was used to impute missing data in both the development cohort and the validation cohort, with 10 imputations generated for the development and validation datasets. Two separate and independent imputation procedures were used, one for model development and one for model validation. The imputation models included all model covariates within each dataset, along with the Nelson-Aalen estimator for the cumulative baseline cause specific hazards for falls and for the competing event of death, and binary event indicators for each of these possible event types.^31^ ^32^ When information was missing on the diagnosis of comorbidities or prescribed drugs, it was assumed that no diagnosis or prescription was present. Predictor variables requiring imputation were cholesterol, ethnicity, deprivation score (validation cohort only), smoking status, and alcohol consumption.

Imputations were assessed for consistency by comparing density plots, histograms, and summary statistics across imputations and back to the complete values. The model coefficients and predictive performance measures were then estimated in each imputed dataset separately, before being combined across imputations using Rubin’s rules.^33^

### Model development

Researchers at the University of Oxford (CK, JPS) conducted the model development and apparent validation. Multivariable prediction models were fitted in each imputed dataset using a Fine-Gray subdistribution hazard model, taking into account the competing risk of death by other causes.^34^ The aim of accounting for the competing risk in this way was to avoid overestimation of the predicted probabilities of falls as defined in the Fine-Gray paper.^34^ ^35^ Predictor effects in the model are reported as subdistribution hazard ratios with 95% confidence intervals, and the post-estimation baseline cumulative incidence for falls was estimated using a Breslow type estimator.^34^ Analyses were undertaken using the *fastcmprsk* package in RStudio.^36^ Automated variable selection methods were not used, since the variables were all predetermined based on the literature and expert opinion, and given the large sample size would result in nearly all predictors having a statistically significant association with the outcome, regardless of effect size. To ensure a parsimonious model, we excluded variables with little or no association in multivariable analysis before fitting the final model.

Fractional polynomial terms were examined to identify the best fitting functional form of all continuous variables.^37^ Fractional polynomials were identified separately within each imputed dataset, and we selected the most consistent transformation across the imputations, choosing lower order fractional polynomial terms whenever possible for the sake of parsimony. We then forced the selected fractional polynomial format for each continuous variable into the model for all imputations to ensure consistency in coefficient estimation.

Interactions between age, sex, and antihypertensive treatments were considered but excluded from the model development owing to problems with stability or convergence, or for the sake of parsimony.

We examined the Schoenfeld residuals to check the proportional hazards assumption for each predictor.^38^

### Apparent validation using development data

Observed outcome probabilities were defined using pseudo values: jack-knife estimators representing an individual’s contribution to the cumulative incidence function for falls, accounting for competing risk, calculated by the Aalen–Johansen method. Pseudo values were generated separately in 50 groups by linear predictor value, for stability, and to account for the competing risk of death and non-informative right censoring.^39^ ^40^

The model’s apparent calibration performance was assessed using calibration plots comparing the observed to predicted risks at one, five, and 10 years. The calibration plots were produced using observed pseudo values and included a smooth (non-linear) calibration curve to show apparent calibration across the spectrum of predicted risks,^41^ with 95% confidence intervals. Plots were generated in each imputed dataset separately and were checked for consistency across imputations. A single, representative example is reported.

When plots showed miscalibration, we recalibrated the original Fine-Gray model separately at each time point by fitting a generalised linear equation with a logit link function directly to the observed pseudo values in the development dataset. The linear predictor from the original model was the only variable included in the recalibration model, which allowed for a non-linear recalibration effect using fractional polynomials.

### External validation

**Fig 1:**
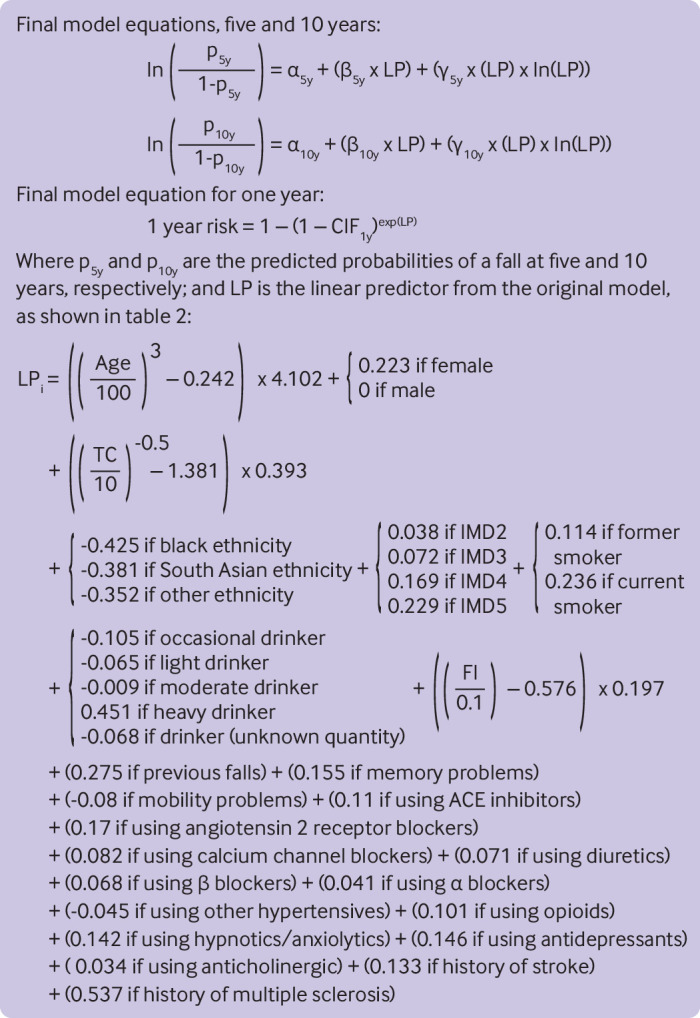
Final model equations for predicting risk of falls at one, five, and 10 years in patients with an indication for hypertensive treatment. Age is measured in years. Ln=natural logarithm; IMD2-IMD5=indices of multiple deprivation; TC=total cholesterol; FI=electronic frailty index. The full algorithm code (including the α, β, γ, and CIF values) is freely available for research use and can be downloaded at https://process.innovation.ox.ac.uk/software/

Researchers at Keele University (LA, KIES, RDR) conducted the external validation of the prediction model, independent of the model development team. The prediction model algorithms presented in figure 1 (both the original and the final) were applied to each individual in the external validation cohort to give the predicted probabilities of experiencing a fall within one, five, and 10 years, taking account of the competing risk of death by other causes.^42^ Model calibration was assessed through comparison of predicted probabilities to observed pseudo values, estimated using jack-knife estimators representing an individual’s contribution to the cumulative incidence function for falls, accounting for competing risks, calculated by the Aalen–Johansen method in the external validation cohort.

Predictive performance was quantified by calculating the observed to expected ratio, Harrell’s C statistic, Royston’s D statistic with its associated R^2^ statistic,^43^ each applied to the same pseudo values as above, and by using calibration plots and curves. Calibration plots were generated separately in each imputed dataset and checked for consistency (one illustrative example is shown for each model). All measures were calculated in each imputed dataset separately and, when appropriate, combined across imputations using Rubin’s rules. When Rubin’s rules did not apply (eg, when the posterior distribution was not expected to be normal), performance was summarised across imputations using the median and interquartile range.^44^

Heterogeneity in model performance across different general practices was assessed using a random effects meta-analysis, using restricted maximum likelihood estimation, given that the case mix and incidence of falls were expected to vary between practices (see extended methods in the supplementary material).^45^ The observed to expected ratio was pooled across practices on the natural log scale, the C statistic on the logit scale (with the standard errors of logit C calculated using the delta method), and the D statistic on its original scale.^46^ ^47^ Pooled estimates are reported with prediction intervals to give an indication of expected model performance in a new general practice.

Clinical utility was assessed by plotting the one year, five year, and 10 year risk of falls against the 10 year risk of cardiovascular disease, calculated using the Qrisk2 algorithm.^22^ Clinical utility was also examined using net benefit analysis, where the harms and benefits of using a model to guide treatment decisions were offset to assess the overall consequences of using the STRATIFY-Falls prediction models for clinical decision making.^48^ The original and final models were compared with one another at five and 10 years and with model blind methods of introducing falls prevention measures (which may include deprescribing) for all patients, or not introducing falls prevention measures (starting or continuing treatment) for all patients, regardless of falls risk. We assessed net benefit across the full range of possible threshold probabilities, with a falls risk above 10% at 10 years specified a priori as being a threshold of clinical interest, to align with current thresholds for an individual’s risk of cardiovascular disease.^49^

The same external validation methods as described earlier were employed in subgroups by age (<65 years, ≥65 years), sex (women, men), and ethnicity (white, black, South Asian, other), to assess the models’ predictive performance in these clinically relevant groups.

### Patient and public involvement

This study was developed and conducted with the help of our patient and public advisor Margaret Ogden. As a member of our study advisory group, they commented on the study protocol and have been present in all team meetings discussing results and reporting. We also held a focus group with several older adults during the study to discuss broader themes related to drugs for cardiovascular disease prevention and adverse events, which informed the interpretation of this work.

## Results

### Study population characteristics

**Fig 2:**
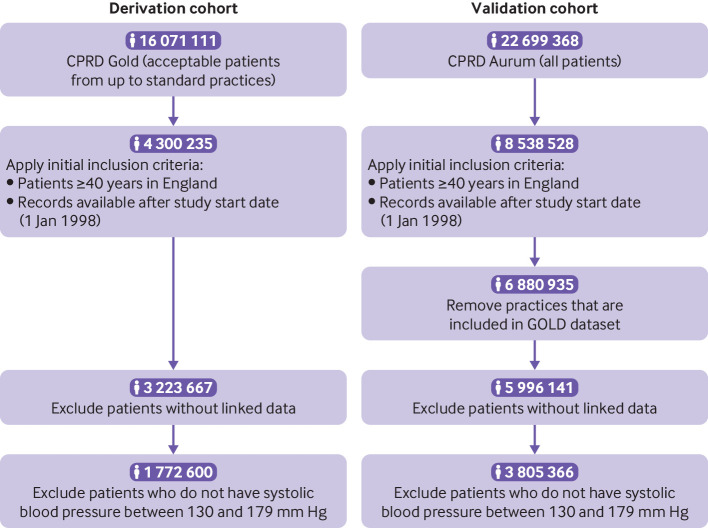
Flow of participants through study. CPRD=Clinical Practice Research Datalink

Figure 2 shows the flow of study participants for both the development cohort and the validation cohort. A total of 1 772 600 patients were included in the model development cohort (CPRD GOLD), with a mean age of 59 years (standard deviation (SD) 13 years) and a mean systolic blood pressure of 144 mm Hg (SD 12 mm Hg) at study inclusion (table 1). The 10 year prevalence of falls was 3.5% (n=62 691), with 10.3% of patients (n=181 731) experiencing death by other causes before any fall occurred, and a median follow-up of 6.2 years (interquartile range (IQR) 2.6-10 years) across the cohort.

**Table 1:** Descriptive statistics for model development and validation cohorts, in full cohorts and stratified by outcome type at 10 years. Values are numbers (percentages) unless stated otherwise

Variables	Development cohort				Validation cohort		
	Total (n=1 772 600)	Falls (n=62 691)	Mortality (n=181 731)		Total (n=3 805 366)	Falls (n=206 956)	Mortality (n=334 552)
Mean (SD) age (years)	59.4 (13.2)	73.6 (12.7)	74.3 (12.0)		58.6 (13.3)	72.8 (12.7)	73.1 (12.3)
Women	921 853 (52)	39 955 (64)	91 676 (50)		1 959 489 (52)	134 945 (65.2)	165 689 (49.5)
Systolic blood pressure (mm Hg)	143.5 (11.9)	146.3 (12.7)	146.9 (12.8)		143.8 (12.3)	147.2 (13.2)	147.7 (13.3)
Diastolic blood pressure (mm Hg)	83.8 (9.6)	81.6 (10.0)	81.7 (10.0)		83.9 (9.8)	81.9 (10.2)	82.0 (10.3)
Cholesterol (mmol/L)	5.3 (1.1)	5.2 (1.2)	5.2 (1.2)		5.5 (1.2)	5.4 (1.3)	5.4 (1.3)
Missing	868 461 (48.9)	32 661 (52)	104 094 (59)		1 839 116 (48.3)	109 708 (53.0)	195 390 (58.4)
Ethnicity:							
White	734 149 (41)	59 608 (95)	105 077 (40.5)		2 041 505 (54)	194 311 (93.9)	206 384 (61.7)
Black	10 799 (0.6)	339 (0.54)	826 (0.45)		115 279 (3)	2239 (1.1)	4019 (1.2)
South Asian	14 799 (0.8)	505 (0.81)	991 (0.55)		94 485 (3)	2449 (1.2)	3673 (1.1)
Other	15 731 (0.9)	587 (0.94)	1229 (0.68)		832 614 (22)	3442 (1.7)	21458 (6.4)
Missing	997 122 (56)	1652 (2.6)	73 608 (57.8)		721 483 (19)	4515 (2.2)	99 018 (29.6)
Index of multiple deprivation score:							
1	420 765 (23.7)	12 624 (20.2)	35 529 (19.6)		790 311 (20.8)	41 786 (20.2)	66 606 (19.9)
2	406 775 (22.9)	13 429 (21.4)	39 652 (21.8)		732 246 (19.2)	41 820 (20.2)	68 147 (20.4)
3	376 765 (21.3)	13 239 (21.1)	39 279 (21.6)		684 288 (18)	40 665 (19.7)	67 130 (20.1)
4	313 595 (17.7)	12 031 (19.2)	35 183 (19.4)		630 482 (16.6)	40 383 (19.5)	65 342 (19.5)
5	254 700 (14.4)	11 317 (18.1)	31 909 (17.6)		597 180 (15.7)	42 141 (20.4)	67 024 (20.0)
Missing	0 (0)	0 (0)	0 (0)		370 859 (9.7)	161 (0.1)	303 (0.1)
Smoking status:							
Non-smoker	847 205 (48)	29 500 (47.1)	74 646 (41)		1 475 708 (39)	77 990 (37.7)	109 249 (32.7)
Former smoker	471 005 (27)	17 440 (27.8)	50 884 (28)		1 236 061 (33)	39 087 (18.9)	75 081 (22.4)
Current smoker	363 440 (21)	10 720 (17.1)	38 478 (21.2)		838 404 (22)	66 836 (32.3)	105 363 (31.5)
Missing	90 950 (5)	5031 (8.0)	17 905 (9.9)		255 193 (7)	23 043 (11.1)	44 859 (13.4)
Median (IQR) frailty index score	0.03 (0-0.08)	0.08 (0.06-0.14)	0.08 (0.06-0.14)		0.06 (0.03-0.08)	0.08 (0.06-0.17)	0.08 (0.06-0.17)
Alcohol intake status:							
Non-drinker	289 472 (16)	14 172 (22.6)	37 568 (20.7)		864 865 (23)	59 364 (28.7)	89 537 (26.8)
Occasional drinker	488 289 (28)	15 195 (24.2)	42 645 (23.5)		998 948 (26)	47 088 (22.8)	71 739 (21.4)
Light drinker	239 732 (14)	6472 (10.3)	18 863 (10.4)		696 369 (18)	26 635 (12.9)	44 924 (13.4)
Moderate drinker	179 102 (10)	3891 (6.2)	12 926 (7.1)		246 468 (7)	9378 (4.5)	17 491 (5.2)
Heavy drinker	22 760 (1.3)	891 (1.4)	2336 (1.3)		74 005 (2)	5124 (2.5)	6845 (2.1)
Unknown amount	291 649 (16)	9962 (15.9)	165 132 (14.4)		237 464 (6)	9631 (4.7)	12 117 (3.6)
Missing	216 596 (15)	12 108 (19.3)	41 261 (22.7)		687 247 (18)	49 736 (24)	91 899 (27.5)
Risk factors:							
Previous falls	108 745 (6)	10 514 (16.8)	22 459 (12.4)		140 886 (3.7)	21 697 (10.5)	25 124 (7.5)
Memory problems	28 276 (1.6)	3860 (6.2)	10 556 (5.8)		99 264 (2.6)	15 996 (7.7)	28 636 (8.6)
Mobility problems	20 425 (1.2)	2462 (3.9)	7347 (4.0)		85 675 (2.3)	13 999 (6.8)	22 928 (6.9)
Stroke	44 339 (2.5)	4320 (6.9)	14 167 (7.8)		111 462 (2.9)	15 704 (7.6)	26 703 (8)
Multiple sclerosis	6367 (0.4)	300 (0.5)	798 (0.4)		11 328 (0.3)	975 (0.5)	1373 (0.4)
Antihypertensive drugs:							
ACE inhibitors	219 506 (12)	12 039 (19.2)	38 096 (20.9)		478 778 (13)	38 867 (18.8)	67 787 (20.3)
Angiotensin 2 receptor blockers	59 075 (3)	3167 (5.1)	7628 (4.2)		136 926 (4)	11 018 (5.3)	14 308 (4.3)
α blockers	34 338 (2)	2088 (3.3)	6794 (3.7)		68 131 (2)	6335 (3.1)	11 388 (3.4)
β blockers	216 122 (12)	10 885 (17.4)	31 341 (17.3)		461 329 (12)	36 317 (17.6)	59 019 (17.6)
Calcium channel blockers	193 141 (11)	11 570 (18.5)	35 859 (19.7)		426 151 (11)	37 590 (18.2)	63 764 (19.1)
Diuretics	180 065 (10)	10 706 (17.1)	29 783 (16.4)		397 980 (11)	36 418 (17.6)	55 934 (16.7)
Other antihypertensives	10 784 (0.6)	400 (0.8)	1594 (0.9)		19 235 (1)	1437 (0.7)	2471 (0.7)
Other drugs:							
Opioids	553 344 (31)	26 060 (41.6)	69 496 (38.2)		1 213 876 (32)	84 108 (40.6)	121 303 (36.3)
Hypnotics and anxiolytics	376 885 (21)	17 703 (28.2)	48 636 (26.8)		750 584 (20)	52 854 (25.5)	78 627 (23.5)
Antidepressants	383 647 (21)	17 159 (27.4)	42 767 (23.5)		793 690 (21)	52 820 (25.5)	71 452 (21.4)
Anticholinergics	207 345 (11)	11 085 (17.7)	29 384 (16.2)		388 513 (10)	31 542 (15.2)	46 255 (13.8)
Median (IQR) follow-up (years)	6.2 (2.6-10)	4.3 (1.8-7.0)	3.7 (1.6-6.3)		6.7 (2.7-10)	4.3 (1.9-7.1)	3.8 (1.6-6.5)

ACE=angiotensin converting enzyme; IQR=interquartile range; SD=standard deviation.

In total, 3 805 366 patients were included in the validation cohort, with 206 956 (5.4%) experiencing fall events during 10 year follow-up. A further 334 552 (8.8%) patients died during follow-up from unrelated causes, before any fall occurred. Median follow-up time in the validation cohort was 6.7 years (IQR 2.7-10 years). Total cholesterol level was missing in 48% of participants, and ethnicity data were more complete in the validation cohort than development cohort (81% *v* 44% complete data).

### Model development

The original model consisted of 24 predictors, after the exclusion of variables with little or no association in multivariable analysis (table 2). Compared with men, women were more likely to experience a fall during follow-up (subdistribution hazard ratio 1.25, 95% confidence interval 1.23 to 1.27). Increasing age, white ethnicity, and being a smoker, a heavy drinker, or more deprived were predictors associated with an increased risk of falls (table 2). Increasing frailty was one of the strongest predictors of falls, with an increased falls risk of 22% for about every four deficits accrued (1.22, 1.20 to 1.23). Of the previous medical conditions examined, the strongest predictors of falls were having a history of falls (1.32, 1.29 to 1.35) and multiple sclerosis (1.71, 1.51 to 1.94). Drugs most strongly associated with falls were angiotensin 2 receptor blockers (1.19, 1.15 to 1.23), antidepressants (1.16, 1.13 to 1.18), hypnotics and anxiolytics (1.15, 1.13 to 1.18), angiotensin converting enzyme inhibitors (1.12, 1.10 to 1.14), and opioids (1.11, 1.08 to 1.13). To ensure a parsimonious final model, systolic and diastolic blood pressure, BMI, activity limitation, syncope, and cataract were excluded from the model owing to a lack of association with falls risk. No violations of the proportional hazards assumption were detected.

**Table 2:** Prediction model for falls. Values are subdistribution hazard ratios and 95% confidence intervals

Predictors	Full case analysis (n=358 207)	Multiple imputation model (n=1 772 600)
Age	30.1 (27.7 to 32.7)	60.46 (57.87 to 63.17)
Sex (women)	1.32 (1.28 to 1.35)	1.25 (1.23 to 1.27)
Total cholesterol	1.55 (1.44 to 1.67)	1.48 (1.36 to 1.61)
Ethnicity:		
White	Reference	Reference
Black	0.68 (0.59 to 0.79)	0.65 (0.58 to 0.74)
South Asian	0.67 (0.60 to 0.75)	0.68 (0.61 to 0.77)
Other	0.66 (0.59 to 0.74)	0.70 (0.63 to 0.78)
Index of multiple deprivation score:		
1	Reference	Reference
2	1.05 (1.00 to 1.09)	1.04 (1.01 to 1.07)
3	1.06 (1.02 to 1.12)	1.07 (1.05 to 1.10)
4	1.14 (1.01 to 1.19)	1.18 (1.15 to 1.21)
5	1.23 (1.18 to 1.29)	1.35 (1.31 to 1.39)
Smoking status:		
Non-smoker	Reference	Reference
Former smoker	1.06 (1.04 to 1.09)	1.12 (1.10 to 1.14)
Current smoker	1.26 (1.22 to 1.31)	1.27 (1.24 to 1.30)
Alcohol intake status:		
Non-drinker	Reference	Reference
Occasional drinker	0.87 (0.84 to 0.90)	0.90 (0.85 to 0.95)
Light drinker	0.93 (0.89 to 0.98)	0.94 (0.88 to 1.00)
Moderate drinker	0.99 (0.94 to 1.05)	0.99 (0.93 to 1.06)
Heavy drinker	1.71 (1.55 to 1.87)	1.57 (1.28 to 1.93)
Unknown amount	0.97 (0.95 to 1.02)	0.93 (0.89 to 0.98)
Frailty index score	1.11 (1.09 to 1.14)	1.22 (1.20 to 1.23)
Risk factors:		
History of falls	1.40 (1.35 to 1.46)	1.32 (1.29 to 1.35)
Memory problems	1.25 (1.17 to 1.35)	1.17 (1.12 to 1.21)
Mobility problems	0.99 (0.93 to 1.07)	0.92 (0.87 to 0.98)
Stroke	1.28 (1.22 to 1.34)	1.14 (1.11 to 1.18)
Multiple sclerosis	1.48 (1.23 to 1.78)	1.71 (1.51 to 1.94)
Antihypertensive drugs:		
ACE inhibitors	1.04 (1.01 to 1.07)	1.12 (1.10 to 1.14)
Angiotensin 2 receptor blockers	1.07 (1.02 to 1.12)	1.19 (1.15 to 1.23)
α blockers	1.00 (0.95 to 1.06)	1.04 (1.02 to 1.06)
β blockers	0.97 (0.96 to 1.00)	1.07 (1.02 to 1.12)
Calcium channel blockers	0.99 (0.97 to 1.03)	1.08 (1.06 to 1.11)
Diuretics	0.98 (0.95 to 1.01)	1.07 (1.05 to 1.10)
Other antihypertensives	1.08 (0.97 to 1.21)	0.96 (0.88 to 1.04)
Other drugs		
Opioids	1.10 (1.07 to 1.13)	1.11 (1.08 to 1.13)
Hypnotics and anxiolytics	1.04 (1.00 to 1.07)	1.15 (1.13 to 1.18)
Antidepressants	1.14 (1.10 to 1.18)	1.16 (1.13 to 1.18)
Anticholinergics	1.11 (1.06 to 1.14)	1.03 (1.02 to 1.05)

ACE=angiotensin converting enzyme.

Variable transformations: Age=((age/100)^3)−0.242; cholesterol=((cholesterol/10)^−0.5)−1.381; frailty index=(frailty index/0.1)−0.576.

### Internal validation and recalibration using pseudo values

**Fig 3:**
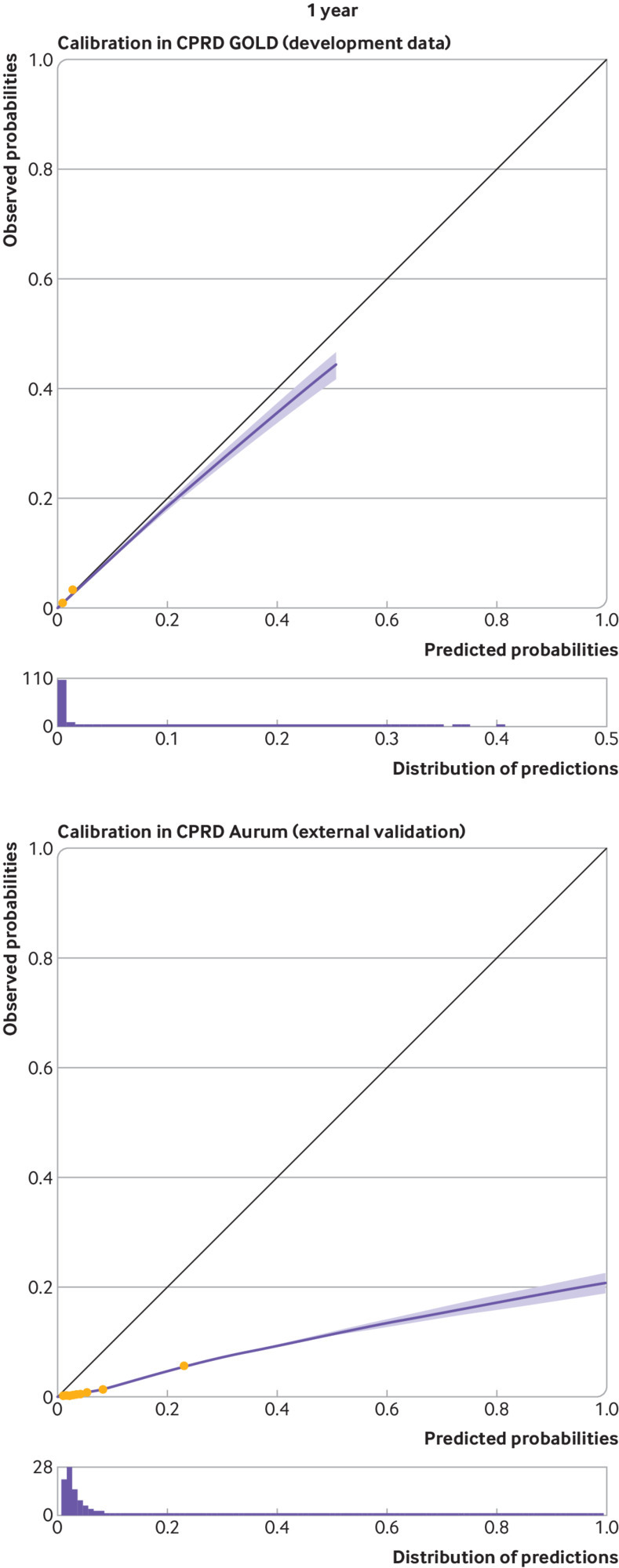
Calibration curves for apparent performance of the final STRATIFY-Falls model in CPRD GOLD at one year, and calibration on external validation in CPRD Aurum at one year. Groups represent 10ths of linear predictor, as created between deciles. Histogram shows distribution of predicted probabilities. The model is not recalibrated to pseudo values in the development data. CPRD=Clinical Practice Research Datalink; STRATIFY=STRAtifying Treatments In the multi-morbid Frail elderly

**Fig 4:**
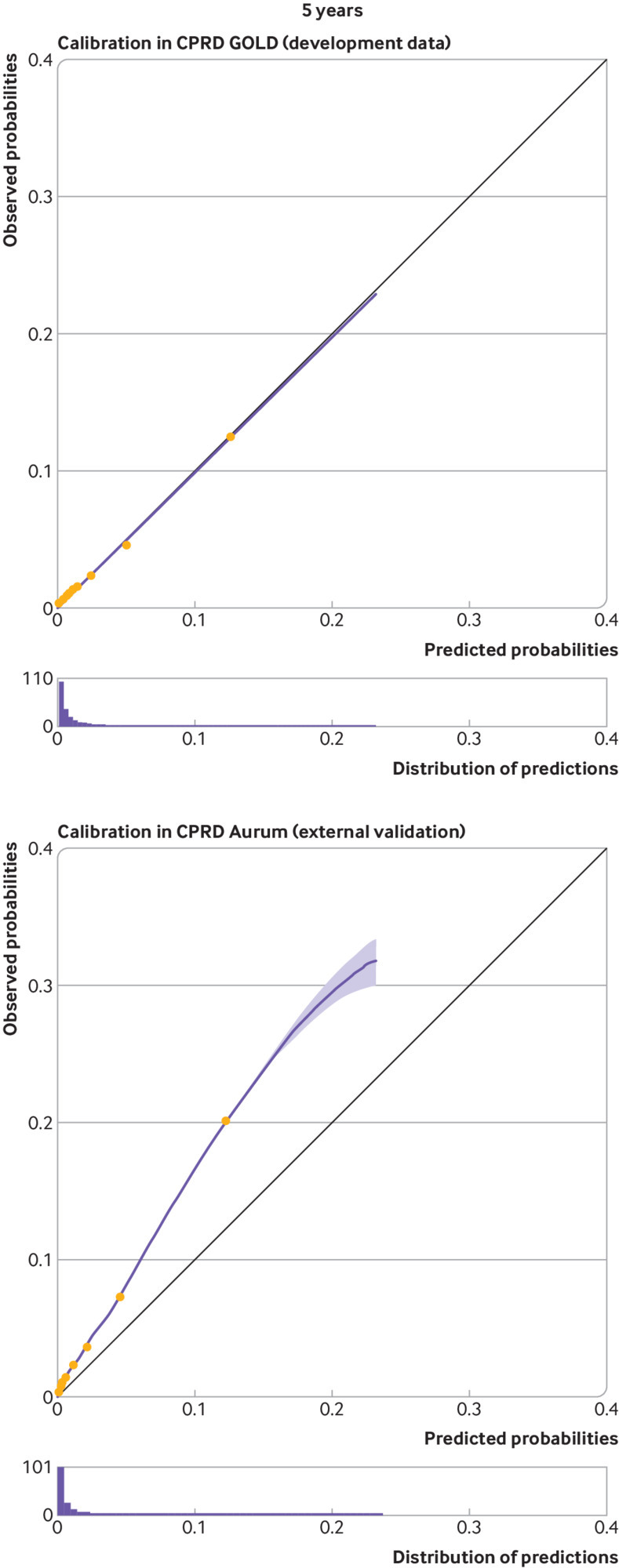
Calibration curves for apparent performance of the final STRATIFY-Falls model in CPRD GOLD at five years, and calibration on external validation in CPRD Aurum at five years. Groups represent 10ths of linear predictor, as created between deciles. Histogram shows distribution of predicted probabilities. CPRD=Clinical Practice Research Datalink; STRATIFY=STRAtifying Treatments In the multi-morbid Frail elderlY

**Fig 5:**
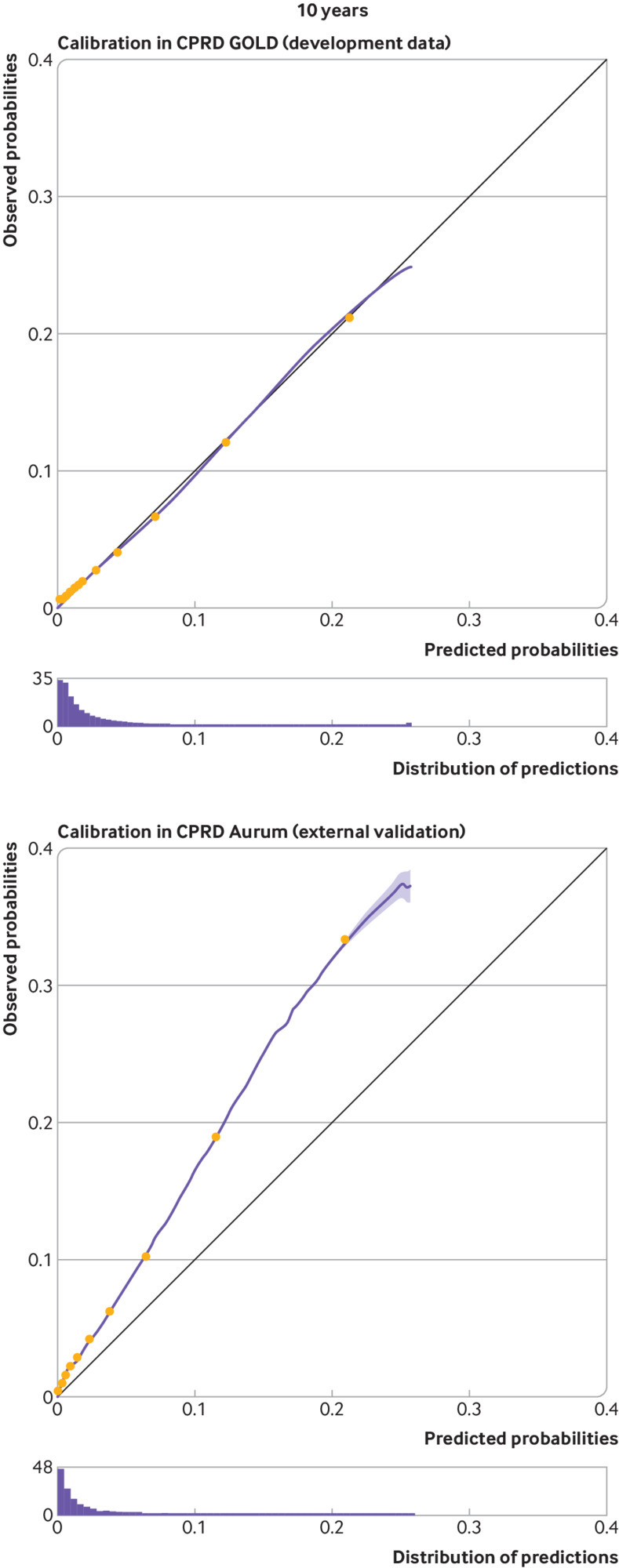
Calibration curves for apparent performance of the final STRATIFY-Falls model in CPRD GOLD at 10 years, and calibration on external validation in CPRD Aurum at 10 years. Groups represent 10ths of linear predictor, as created between deciles. Histogram shows distribution of predicted probabilities. CPRD=Clinical Practice Research Datalink; STRATIFY=STRAtifying Treatments In the multi-morbid Frail elderlY

At five and 10 years, apparent calibration plots in the model development data showed significant miscalibration, with under-prediction for patients with a low predicted risk and substantial over-prediction for those with a high predicted risk (see supplementary figure S3.1). We therefore recalibrated the original model to the observed pseudo values and this improved apparent calibration (in the model development data) considerably (fig 4 and fig 5). Apparent calibration of the original model at one year was good, therefore recalibration was not required (see fig 3).

### External validation

#### Predictive performance

Upon external validation, the original model showed excellent discrimination (table 3) but poor calibration (see supplementary figure S3.1), with considerable heterogeneity across general practices (see supplementary figure S3.2). Recalibration of the model corrected miscalibration in the model development cohort, but under-prediction of risk was still present in the validation cohort (fig 3, fig 4, and fig 5). This miscalibration was less extreme than that of the original model, in the narrower range of predicted probabilities between 0 to 0.2. On average, the recalibrated model showed a pooled observed to expected ratio at 10 years of 1.839 (95% confidence interval 1.811 to 1.865, 95% prediction interval 1.284 to 2.638), suggesting that the observed incidence of falls would be around 84% (relatively) higher than expected when using the model to generate predictions. Under-prediction of 10 year falls risk was consistent across all subgroups, with the exception of the “other ethnicity” group, where both the falls incidence and the observed to expected ratio were considerably lower than in the full population (see extended results in supplementary material section 2.2).

**Table 3:** Predictive performance statistics of the falls prediction models on external validation in Clinical Practice Research Datalink Aurum

Statistics	1 year		5 years			10 years	
	Original model		Original model	Pseudo value recalibration		Original model	Pseudo value recalibration
**Observed to expected ratio**							
Pooled effect size (95% CI)	0.162 (0.158 to 0.166)		1.702 (1.674 to 1.730)	1.906 (1.874 to 1.939)		1.682 (1.657 to 1.707)	1.839 (1.811 to 1.865)
Prediction interval	0.090 to 0.289		1.116 to 2.586	1.246 to 2.915		1.139 to 2.484	1.284 to 2.638
τ^2^	0.089 (0.080 to 0.099)		0.046 (0.042 to 0.052)	0.0479 (0.043 to 0.054)		0.038 (0.035 to 0.043)	0.0342 (0.031 to 0.038)
**C statistic**							
Pooled effect size (95% CI)	0.866 (0.862 to 0.869)		0.843 (0.841 to 0.844)	0.843 (0.841 to 0.844)		0.833 (0.832 to 0.835)	0.833 (0.831 to 0.835)
Prediction interval	0.794 to 0.915		0.789 to 0.881	0.789 to 0.881		0.789 to 0.870	0.789 to 0.870
τ^2^	0.068 (0.056 to 0.083)		0.026 (0.023 to 0.030)	0.026 (0.023 to 0.030)		0.022 (0.019 to 0.025)	0.022 (0.019 to 0.025)
**D statistic**							
Pooled effect size (95% CI)	2.160 (1.987 to 2.333)		1.903 (1.754 to 2.051)	1.894 (1.746 to 2.042)		1.643 (1.515 to 1.771)	1.597 (1.472 to 1.721)
Prediction interval	1.99 to 2.33		1.75 to 2.05	1.75 to 2.04		1.51 to 1.77	1.47 to 1.72
τ^2^	0.000 (0.000 to 0.039)		0.000 (0.000 to 0.023)	0.000 (0.000 to 0.022)		0.000 (0.0000 to 0.0168)	0.000 (0.000 to 0.016)
**Royston and Sauerbrei’s R^2^**							
Range	0 to 86.0		28.0 to 91.4	25.9 to 91.4		21.3 to 91.4	21.6 to 91.4
Median (IQR)	58.1 (52.3 to 62.2)		47.4 (43.5 to 51.8)	47.3 (43.2 to 51.7)		39.9 (36.4 to 43.8)	38.6 (35.4 to 42.4)
Mean (SD)	56.5 (0.10)		47.9 (0.07)	47.7 (0.07)		40.8 (0.07)	39.4 (0.07)

CI=confidence interval; IQR=interquartile range; SD=standard deviation.

**Fig 6:**
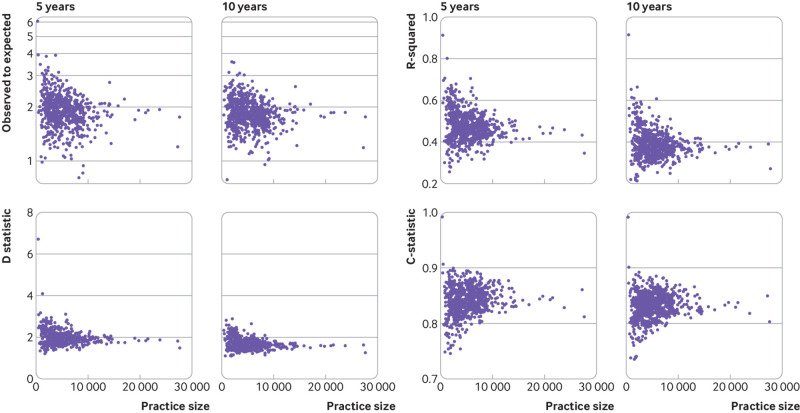
Performance variability of the final STRATIFY-FALLS model on external validation across general practices, with observed to expected ratio, R^2^ statistic, D statistic, and C statistic. STRATIFY=STRAtifying Treatments In the multi-morbid Frail elderlY

The ordering of participants’ predicted probabilities altered only slightly on recalibration; thus discriminative ability of the recalibrated models remained excellent at each of the analysis time points, with C statistics of 0.843 (95% confidence interval 0.841 to 0.844, 95% prediction interval 0.789 to 0.881) at five years, and 0.833 (0.831 to 0.835, 95% prediction interval 0.789 to 0.870) at 10 years, and D statistic values of 1.894 (1.746 to 2.042, 95% prediction interval 1.75 to 2.04) at five years, and 1.597 (1.472 to 1.721, 95% prediction interval 1.47 to 1.72) at 10 years (table 3). Model performance varied more among smaller practices, with more consistent performance seen as practice size increased (fig 6).

The model’s discriminative ability at 10 years was consistent across age and sex subgroups (see supplementary tables S2.1 and S2.2). The pooled C statistic was lowest in those of white ethnicity (0.796, 95% confidence interval 0.793 to 0.798) and highest among those of other ethnicity (0.834, 0.830 to 0.839) (see supplementary table S2.3).

#### Clinical utility analysis

**Fig 7:**
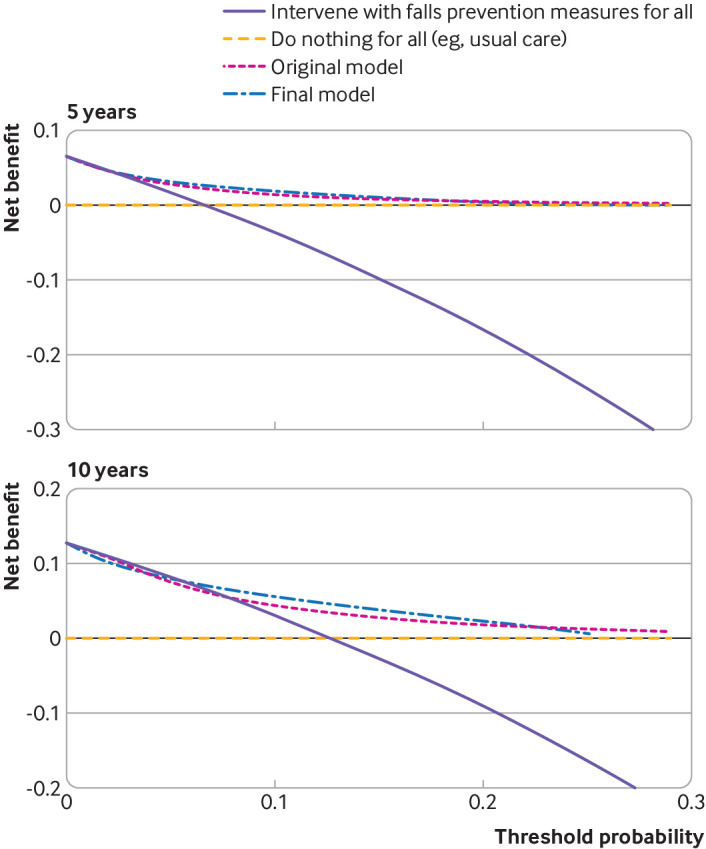
Decision curve analysis showing net benefit of using prediction models across different threshold probabilities for assigning treatment

Net benefit and decision curve analysis of the original and recalibrated models indicated potential clinical utility at five and 10 years around the predefined threshold of 10% (fig 7). At 10 years, basing clinical management decisions on predicted probabilities of falls yielded a benefit over the two strategies of introducing falls prevention measures (which may include deprescribing) for all and not introducing falls prevention measures (starting or continuing treatment) for all patients, when using a treatment decision threshold of 7% or higher from the original model, or a treatment decision threshold of 6% or higher from the final recalibrated model. Thus, for either model, when using our prespecified treatment decision cut-off of 10% risk of falls at 10 years, we would expect a benefit to patients over and above model blind treatment strategies (usual care). This treatment decision threshold of 10% showed a net benefit in all subgroups except other ethnicity, where a cut-off of at most 3% was required for the model to be superior to usual care for all (see supplementary figure S2.6). In the analysis at five years, using a treatment decision threshold of 3% risk or higher gave a net benefit above starting or continuing treatment for all, for both models.

**Fig 8:**
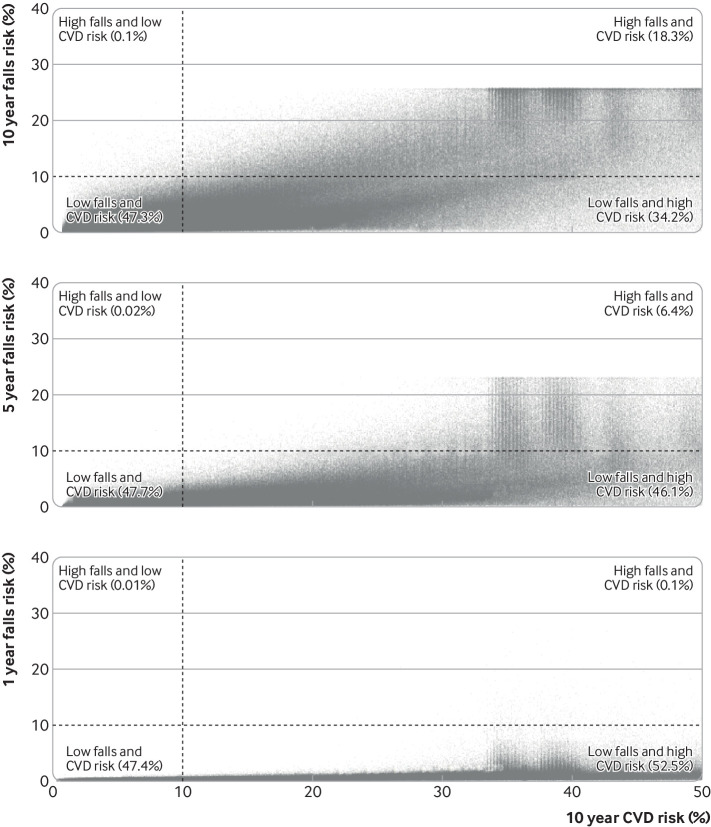
Comparison of 10 year cardiovascular disease risk (Qrisk2) and fall risk in Clinical Practice Research Datalink GOLD dataset. High risk for both conditions was defined as a risk >10%. CVD=cardiovascular disease

In analyses comparing the risk of falls with the risk of cardiovascular disease in CPRD GOLD, 1725 (0.1%) patients had a high risk of falls (>10%) but low risk of cardiovascular disease (<10%) at 10 years (fig 8). A further 324 884 (18.3%) patients were classified as high risk of both, and 607 228 (34.2%) had a low falls risk but high risk of cardiovascular disease.

## Discussion

### Principal findings

We developed and externally validated a clinical prediction model to determine an individual’s risk of experiencing a fall resulting in hospital admission or death within 10 years of being indicated for antihypertensive treatment (owing to raised blood pressure readings). The model incorporates routinely recorded information, including a history of previous falls, multiple sclerosis, heavy alcohol consumption, high deprivation score, and prescribed drugs, which were all strong predictors of subsequent falls, conditional on the other model variables.

The final recalibrated model showed good discrimination upon external validation, suggesting that it can help distinguish those at a higher risk of falling, which may improve how doctors identify patients who might benefit from targeted fall prevention strategies, including multifactorial or exercise based interventions,^50^ and drug reviews including deprescribing. Calibration performance of the prediction model was inconsistent across the development and validation datasets, with miscalibration leading to under-prediction of fall risk across the full range of predicted probabilities. Nevertheless, such under-prediction of risk may be deemed acceptable if the model is intended to inform whether treatment should be stopped to avoid adverse effects—particularly if the treatment in question also carries benefits. Indeed, the clinical utility analysis showed that at risk thresholds around 10%, the net benefit of the model is higher than for other strategies currently employed in usual care.

### Strengths and limitations of this study

Strengths of this work include the large, population based cohorts used, incorporating routinely collected patient data that have been shown to be representative of the patients across England, suggesting that the findings could be generalised across this (or a similar) population.^20^ ^21^ Analyses accounted for the competing risk of death in both model development and external validation, ensuring that falls risk was not over-estimated. This is particularly important in individuals with frailty and multiple long term conditions, where an over-estimation of falls risk might preclude prescription of antihypertensive drugs in those who could still derive benefit from continued treatment. This analysis method is superior to most prediction models in widespread use, which do not take into account competing risks.^22^ In these models, the stated risk of an event (cardiovascular disease, for example) is by design too high, as the actual risk of an event would be diminished by death from other (eg, non-cardiovascular) causes, particularly in older people.^35^

All data were derived from routine electronic health records, including the outcome definition of falls. Such a definition might not capture all events that could be included in the ProFaNE (Prevention of Falls Network Europe) consensus definition of a fall (ie, an unexpected event in which the participants come to rest on the ground, floor, or lower level),^51^ and therefore the model results should be interpreted in this context. It is possible that some of these fall events were not reported or captured correctly within the electronic health record, therefore potentially underestimating the incidence of falls, which could have affected the performance of the model.

Assessments of the models’ predictive performance were conducted across a range of general practices, with different case mix and outcome prevalence, giving an indication of the expected spread of performance across a range of subpopulations. Model performance varied more among smaller practices, with more consistent performance seen as practice size increased. This reflects the increased uncertainty in the estimation of the predictive performance measures in practices of low sample sizes, many of which individually would have failed to meet the required sample size for this external validation. Prediction intervals from meta-analyses across general practices give an indication of how well our falls models would be expected to perform in new practices, helping to inform decisions on implementation in practice. In the present study, the prediction intervals were relatively narrow across a range of performance statistics, suggesting that the models would perform similarly in a new practice from a similar population.

All variables included in our model were predetermined based on the literature, although we did choose to exclude some variables at the model development stage that had exhibited a negligible effect on the outcome. These variables were excluded because they did not contribute substantially to model predictions and served to unnecessarily increase the complexity of the equation. We did not use statistical selection methods such as backwards or forwards elimination, as these can lead to overfitting. Although our approach may have meant that some statistically significant (but clinically insignificant) predictors were excluded from the final model, these exclusions are unlikely to have led to overfitting given the large sample size or been the reason for miscalibration in the external validation.

For these models, we defined binary variables for antihypertensive drugs as any prescription within the year before (and including) the index date, without accounting for any changes to drugs during follow-up. Not allowing for the time varying nature of treatment could potentially affect the observed associations with falls risk, and so too the predicted risks obtained from the model. However, our model is intended to give a prediction for risk of falls over the next 1-10 years, from a particular moment in time, in the context of current care. The latter is important, because, for example, if a patient has low risk, then it means that current care (ie, treatments and monitoring strategies over the next 1-10 years) is likely to be adequate for this individual. In contrast, if an individual’s risk is high, it means that current care is likely insufficient and that additional or alternative approaches are potentially needed.

Calibration performance of the prediction model was inconsistent across the model development and validation datasets. Such miscalibration was surprising, as populations were similar across both datasets for predictor distributions and the incidence of falls and of death (with the exception of self-reported characteristics such as smoking status, alcohol consumption, and ethnicity, which may reflect differences in how these data are captured within the electronic health record systems that underlie these databases). Distributions of the linear predictor were also consistent across the development and validation datasets, suggesting miscalibration could be due to differences in the outcomes or the outcome recording or coding. This is representative of real life, where outcome definitions vary, and both models still exhibited useful discrimination and potential clinical utility across the full population for a range of treatment decision threshold probabilities, although the predicted risk for individuals may be different (miscalibrated) from their actual risk. Indeed, miscalibration was most evident in the 5-10% of patients with the highest predicted risk (those above a threshold of 10%), and in these patients, doctors may interpret the exact predicted risks with caution, even though these patients can still be considered at higher risk.

### Comparison with previous literature

Several prediction models can now estimate an individual’s risk of falls, including those for use in the community. A recent systematic review of development and validation studies identified a total of 72 existing models.^10^ These were typically poorly reported, with only 40 studies (56%) reporting discrimination statistics and seven studies (10%) reporting calibration. Only three models were externally validated. Discrimination was reported with area under the curves of 0.49 to 0.87 for internally validated models and 0.62 to 0.69 for externally validated models. Calibration was moderately good but presented in 10ths of risk across a small range of risk thresholds (eg, 0-10% 52) making it difficult to determine how calibration varied across the full range of predicted probabilities. All studies were deemed at high risk of bias owing to methods of analysis and outcome assessment along with restrictive eligibility criteria.

In contrast, our final model, reported in line with the transparent reporting of a multivariable prediction model for individual prognosis or diagnosis (TRIPOD) guidelines for reporting of clinical prediction models^53^ (see supplementary table S4.3), showed excellent discrimination upon external validation, with an area under the curve of 0.84. It demonstrated reasonable calibration across the low range of predicted risks typically examined by previous risk models (eg, 0-10%) and although miscalibration was present at higher predicted probabilities, there was still clinical utility based on the decision curve analysis. This suggests that the present model is the most promising clinical prediction model for falls available to date, and that it may be effective in identifying individuals at high risk of falls from those in primary care with raised blood pressure.

### Implications for policy and practice

As patients age, their risk of a fall resulting in serious injury and long term disability increases.^4^ Identifying those most at risk is therefore important to enable targeting of fall prevention strategies.^7^ The present model provides primary care doctors with a method of estimating the risk of falls using data routinely available in electronic health records and could have uses beyond predicting falls in patients being considered for antihypertensive treatment.^54^

Among patients aged 40 years and older, with an indication for antihypertensive drugs owing to raised blood pressure, the model was shown to distinguish well those at high risk of falls in the next 1-10 years. Miscalibration was noted, with an under-prediction of risk seen particularly at higher predicted probabilities. Depending on how the model might be used, such under-prediction might be less of a concern—for example, if the model was being used to inform treatment changes only above a certain threshold of predicted risk. In this context, doctors could be confident that the true risk is at least at this threshold, if not higher. Further studies are, however, needed to explore the appropriate thresholds that maximise the model’s clinical utility and cost effectiveness, and to examine whether recalibration is possible in local settings.

The model may also be used to target falls prevention strategies to patients with the highest risk. These strategies might include multifactorial or exercise based interventions,^50^ or review of prescribed drugs, with those drugs likely to increase the risk of falls being considered for deprescribing.^4^ ^18^ Such drug reviews are increasingly being encouraged in routine clinical practice, and the STRATIFY-Falls model may be useful for informing these reviews.^55^ For example, in patients prescribed antihypertensive treatment, the model might be used alongside a cardiovascular risk prediction algorithm to compare the potential for benefit and harm from continued treatment prescription.^26^ ^27^ ^56^ For individuals with a high risk of falls but low risk of cardiovascular disease, a doctor might consider whether new or continued antihypertensive treatment is still appropriate. We examined the prevalence of this scenario in our model development population (fig 8) and identified only a small number of individuals (0.1%) who would be classified in this way, when comparing risks at 10 years. More common, however, were individuals with a low risk of falls but high risk of cardiovascular disease (affecting one in three patients). For these patients, doctors could use the model to illustrate the minimal risk of harm for individuals, potentially improving uptake of, adherence to, and persistence with antihypertensive treatment, which is known to be poor currently.^57^

### Conclusions

The STRATIFY-Falls prediction model helps to identify those at high risk of falls and could be used by doctors wanting to identify patients who might benefit from targeted fall prevention strategies, including multifactorial or exercise based interventions^50^ and drug reviews. Used alongside other prediction tools such as those for cardiovascular risk, such a model could be valuable when used as part of a wider risk assessment for falls prevention.

**What is already known on this topic**

Serious falls are a possible side effect of antihypertensive treatment, which can adversely affect patients’ quality of life and increase the risk of hospital admission, especially in older people with frailty
Existing tools that estimate an individual’s risk of falls have been shown to be at high risk of bias, with only moderate discriminative ability

**What this study adds**

In the present study, a clinical prediction model for the risk of falls for up to 10 years was developed and externally validated, incorporating commonly recorded patient characteristics, comorbidities, and drugs, in patients with an indication for antihypertensive treatment
Upon external validation, the model discriminated well between patients who went on to have a serious fall and those who did not, but calibration indicated under-prediction of risk
Nevertheless, a decision curve analysis suggests the model has clinical utility and so may be useful to identify patients with a high fall risk, who may require closer monitoring or early intervention to prevent future falls

## Data Availability

Data were obtained via a Clinical Practice Research Datalink (CPRD) institutional licence. Requests for data sharing should be made directly to the CPRD. The algorithm is freely available for research use and can be downloaded from https://process.innovation.ox.ac.uk/software/. Code lists used to define variables included in the dataset are available at https://github.com/jamessheppard48/STRATIFY-BP/tree/STRATIFY-Falls

## Web extra

Extra material supplied by authors

## Notes

Contributors: JPS conceived the project and wrote the protocol with FDRH, RJM, RS, and RDR. CK and SLF extracted data for analysis. CK developed the model under supervision of JS and RS. LA validated the model under supervision of RDR and KIES. LA, KIES, and RDR wrote the first draft of the manuscript. All authors revised the manuscript and approved the final version. JPS is the guarantor for this work and accepts full responsibility for the conduct of the study, had access to the data, and controlled the decision to publish. The corresponding author (JPS) attests that all listed authors meet authorship criteria and that no others meeting the criteria have been omitted.

The manuscript’s guarantor (JPS) affirms that the manuscript is an honest, accurate, and transparent account of the study being reported; that no important aspects of the study have been omitted; and that any discrepancies from the study as originally planned (and, if relevant, registered) have been explained.

Dissemination to participants and related patient and public communities: Findings from this study will be press released alongside publication of this manuscript. Social media (eg, Twitter) will be used to draw attention to the work and stimulate debate about its findings. We will also publish a lay summary of our findings on our study website https://www.phc.ox.ac.uk/research/stratified-treatments/studies/stratifying-treatments-in-the-multi-morbid-frail-elderly-stratify-antihypertensives and make the underlying developed algorithms freely available for academic use here: https://process.innovation.ox.ac.uk/software/.

Provenance and peer review: Not commissioned; externally peer reviewed.
