# Development and external validation of a risk prediction model for falls in patients with an indication for antihypertensive treatment: retrospective cohort study

**DOI:** 10.1136/bmj.p2267

**Published:** 2023-10-24

In this paper by Archer and colleagues (*BMJ* 2022;379:e070918; doi:10.1136/bmj-2022-070918, published 8 November 2022) several errors occurred during its preparation.

## Abstract

*Main outcome measures*: The second sentence should state: Model development was conducted using a Fine-Gray approach in data from CPRD GOLD, accounting for the competing risk of death from other causes, with subsequent recalibration at “five and 10 years” using pseudo values.

*Results*: The third sentence should state: Upon external validation, the recalibrated model showed good discrimination, with pooled C statistics of “0.843 (95% confidence interval 0.841 to 0.844) and 0.833 (0.831 to 0.835)” at five and 10 years, respectively.

## Figures 7 and 8

*Figure 7*: The labelling of the decision curves was inadvertently transposed—the top panel shows the results for five years, the bottom panel for 10 years.

*Figure 8*: The authors identified an error in the analysis code used to generate figure 8.

## Results: Clinical utility analysis

The final paragraph should state: In analyses comparing the risk of falls with the risk of cardiovascular disease in CPRD GOLD, “1725 (0.1%)” patients had a high risk of falls (>10%) but low risk of cardiovascular disease (<10%) at 10 years (fig 8). A further “324 884 (18.3%)” patients were classified as high risk of both, and “607 228 (34.2%)” had a low falls risk but high risk of cardiovascular disease.

## Discussion: Implications for policy and practice

The third sentence before the end should state: We examined the prevalence of this scenario in our model development population (fig 8) and identified only “a small” number of individuals “(0.1%)” who would be classified in this way, when comparing risks at 10 years.

The article and typeset pdf have been revised.

