# Supplementary material for: Development and external validation of a risk prediction model for falls in patients with an indication for antihypertensive treatment: retrospective cohort study
Source: BMJ. 2022 Nov 8;379:e070918. doi: 10.1136/bmj-2022-070918 (PMC9641577; doi:10.1136/bmj-2022-070918)
Supplement: Supplementary file 1 — Supplementary information: additional material [file arcl070918.ww.pdf]

Predicting the risk of falls in patients with an indication for antihypertensive treatment: development and external validation of the STRATIFY-Falls prediction model - Supplementary material

Lucinda Archer (0000-0003-2504-2613),[1] *lecturer,* Constantinos Koshiaris*, statistician,*[2] Sarah Lay-Flurrie, *senior statistician*,[2] Kym IE Snell (0000-0001-9373-6591) *senior lecturer,*[1] Richard D Riley, *professor of biostatistics*,[1] Richard Stevens, *associate professor,*[2] Amitava Banerjee, *professor of clinical data science* (0000-0001-8741-3411)[3], Juliet A Usher-Smith, *assistant professor* (0000-0002-8501-2531)[4], Andrew Clegg, *professor of geriatric medicine*[5], Rupert A Payne, *senior lecturer* (0000-0002-5842-4645)[6], FD Richard Hobbs, *Nuffield professor of primary care*[2], Richard J McManus*, professor of primary care research* (0000-0003-3638-028X)[2], James P Sheppard (0000-0002-4461-8756), *associate professor*,[2] on behalf of the STRATIFY investigators*

[1] Centre for Prognosis Research, School of Medicine, Keele University

[2] Nuffield Department of Primary Care Health Sciences, University of Oxford

[3] Institute of Health Informatics, University College London, London, UK

[4] Primary Care Unit, Department of Public Health and Primary Care, University of Cambridge, UK

[5] Academic Unit for Ageing and Stroke Research, Bradford Institute for Health Research, University of Leeds, UK

[6] Centre for Academic Primary Care, Population Health Sciences, University of Bristol, Bristol, UK

*The STRAtifying Treatments In the multi-morbid Frail elderlY (STRATIFY) investigators include the authors and the following:
John Gladman, professor of medicine of older people, School of Medicine, University of Nottingham; Simon Griffin, professor of primary care, Department of Public Health and Primary Care, Primary Care Unit, University of Cambridge; and Margaret Ogden, Patient and Public Involvement advisor.

Corresponding author: James P Sheppard

Email: james.sheppard@phc.ox.ac.uk

Telephone: +44 1865 617192

Address: Nuffield Department of Primary Care Health Sciences, Radcliffe Primary Care Building, Radcliffe Observatory Quarter, University of Oxford, Oxford, OX2 6GG, UK

Contents

1 Extended methods 4

1.1 Sample size 4

1.2 Assessment of model performance across GP surgeries 4

2 Extended results 6

2.1 External validation performance of the original model 6

2.2 Subgroup analyses – falls at 10 years 7

Model performance by age (<65 years, ≥65 years) 7

Model performance by sex (female, male) 9

Model performance by ethnicity 11

3 Further supplementary figures 13

Figure S3.1: Calibration curves for the apparent performance of the original model in CPRD GOLD, and the calibration on external validation in CPRD Aurum. 13

Figure S3.2: Performance variability of the original model on external validation across GP practices, with panels (A) Observed/Expected, (B) $RD2$, (C) D-statistic, and (D) C-statistic 14

Figure S3.3: Falls incidence by practice at 5 and 10 years 15

Figure S3.4: Distribution of linear predictor, grouped by outcome type 16

4 Further supplementary tables 17

Table S4.1: ICD9/10 codes for Fall 17

Table S2: Predictors included in the model development 18

Table S3: TRIPOD checklist 24

5 CPRD Independent Scientific Advisory Committee (ISAC) (protocol number 19_042) - PART 2: PROTOCOL INFORMATION 26

A. Study Title (Max. 255 characters) 26

B. Lay Summary (Max. 250 words) 26

C. Technical Summary (Max. 300 words) 27

D. Outcomes to be measured 28

E. Objectives, Specific Aims and Rationale 28

F. Study Background 28

G. Study Type 30

H. Study Design 30

I. Feasibility counts 30

J. Sample size considerations 30

K. Planned use of linked data (if applicable): 31

L. Definition of the Study population 31

M. Selection of comparison group(s) or controls ... 32
N. Exposures, Outcomes and Covariates ... 32
O. Data/ Statistical Analysis ... 33
P. Plan for addressing confounding ... 36
Q. Plans for addressing missing data ... 36
R. Patient or user group involvement (if applicable) ... 37
S. Plans for disseminating and communicating study results, including the presence or absence of any restrictions on the extent and timing of publication ... 38
T. Limitations of the study design, data sources, and analytic methods ... 39
U. References ... 39
List of Appendices ... 43
Amendments ... 44
Amendment 1 – 19/05/2019 (approved) ... 44
Amendment 2 – 28/06/2021 (approved 05/07/2021) ... 44
Amendment 3 – 25.11.2021 (approved 03/12/2021) ... 46

1 Extended methods

1.1 Sample size

Our pre-specified sample size calculation for model derivation assumed a falls rate of 88.3 per 10,000 person years (52), an expected median follow up of 7 years, an estimate of Nagelkerke's R^2 statistic of 0.15 and a maximum number of 40 predictor parameters in the model. Based on these inputs, a sample size of approximately 15,358 person-years (2,194 participants) was estimated to be required for the development of this prognostic model (23). In our analysis, we in fact considered a total of 44 predictor parameters, which instead required a sample size of approximately 16,898 person-years (2,414 participants). Our development sample size in CPRD GOLD far exceeded this.

For the external validation, our sample size calculation assumed an approximate skew-normal distribution for the model's linear predictor with a mean of 0.41, a variance of 0.8, a skewness parameter of 1, and a kurtosis parameter of 4; an assumed exponential distribution of survival times, with baseline rate parameter 0.0042 to ensure 91% survival at 10 years, and a constant censoring rate with censoring times following an exponential distribution with a rate parameter of 0.096 (to a give a probability of censoring by 10 years of about 85%). This resulted in a sample size of 12,000 patients (experiencing approximately 708 falls) which would be sufficient to target a 95% confidence interval of width 0.2 around the estimate of the calibration slope (24). Once again the sample size in the CPRD Aurum validation cohort far exceeded this.

1.2 Assessment of model performance across GP surgeries

Heterogeneity in model performance across different GP surgeries was assessed using a random effects meta-analysis, using restricted maximum likelihood estimation (REML), given that the case mix and incidence of falls were expected to vary between practices (40). Confidence intervals for pooled estimates were derived using the Hartung-Knapp-Sidik-Jonkman variance correction, to account for any uncertainty in the between-practice

variance (53). Performance estimates were first calculated for each GP practice, within each imputed dataset. Then estimates were combined across imputations, within GP practice by applying Rubin's Rules to combine statistics (and corresponding standard errors) where appropriate. Finally, estimates were combined across practices using meta-analysis to gain estimates of average model performance across all populations (54).

2 Extended results

2.1 External validation performance of the original model

Upon external validation, the original model showed excellent discriminative, with pooled C-statistics of 0.843 (95% CI: 0.841 to 0.844, 95% PI: 0.789 to 0.881) and 0.833 (95% CI: 0.832 to 0.835, 95% PI: 0.789 to 0.870) at 5 and 10 years respectively (Table 3). This was reinforced by corresponding values for the pooled D-statistic of 1.903 (95% CI: 1.754 to 2.051, 95% PI: 1.75 to 2.05) and 1.643 (95% CI: 1.515 to 1.771, 95% PI: 1.51 to 1.77).

The original model showed poor calibration at 5 and 10 years, where the model under-predicted the risk of a fall in those with lower predicted probabilities, while over-predicting the risk in those with higher predicted probabilities (Figure S1, Table 3). The original model also showed overprediction of falls risk at 1 year, across the full range of predicted probabilities (Figure 1).

GP level performance estimates showed considerable heterogeneity for calibration, with under-prediction of falls at both 5 and 10 years evident on average (supplementary figure S2). Variability in estimated O/E is most visible in smaller GP practices, with a lower incidence of falls over the study period. On average across GP practices, the original model showed a pooled O/E ratio at 10 years of 1.682 (95% CI: 1.657 to 1.707, 95% PI: 1.139 to 2.484), suggesting that the observed incidence of falls would be around 68% (relatively) higher than expected when using the model to generate predictions.

2.2 Subgroup analyses – falls at 10 years

Model performance by age (<65 years, ≥65 years)

Figure S2.1: Calibration plots by age group

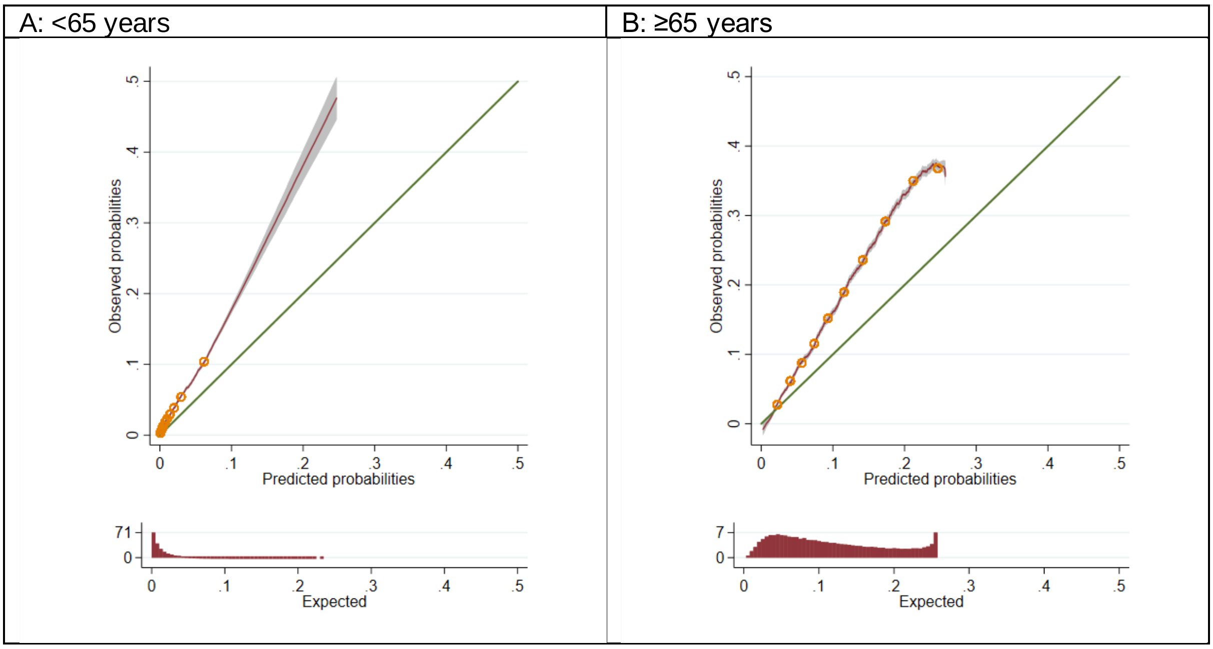

Table S2.1: Predictive performance statistics by age group

	<65 years	≥65 years
**N**	2,592,694	1,212,672
**Falls events (%)**	48,815 (1.9%)	158,141 (13.0%)
**Observed/Expected**		
Pooled effect size (95% CI)	2.098 (2.056 to 2.14)	1.629 (1.603 to 1.654)
Prediction interval	1.259 to 3.490	1.094 to 2.411
Tau^2	0.068 (0.061 to 0.076)	0.040 (0.036 to 0.045)
**C statistic**		
Pooled effect size (95% CI)	0.734 (0.732 to 0.737)	0.732 (0.730 to 0.734)
Prediction interval	0.696 to 0.77	0.681 to 0.777
Tau^2	0.009 (0.008 to 0.012)	0.015 (0.013 to 0.018)
**D statistic**		
Pooled effect size (95% CI)	1.234 (1.133 to 1.336)	0.895 (0.822 to 0.969)
Prediction interval	0.90 to 1.57	0.65 to 1.14
Tau^2	0.027 (0.005 to 0.062)	0.014 (0.001 to 0.034)
**Royston and Sauerbrei's R_D^2**		
Range	0.8 to 78.2	0.2 to 47.7
Median (IQR)	32.5 (27.4 to 38.1)	20.5 (16.9 to 23.8
Mean (SD)	32.8 (9.2)	20.7 (6.5)

Figure S2.2: Decision curve analysis by age group

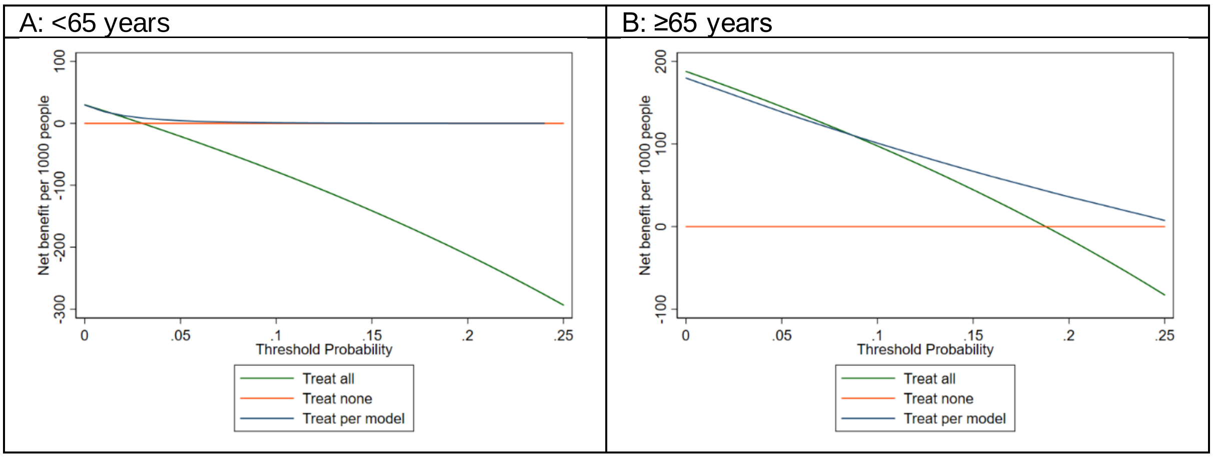

Model performance by sex (female, male)

Figure S2.3: Calibration plots by sex

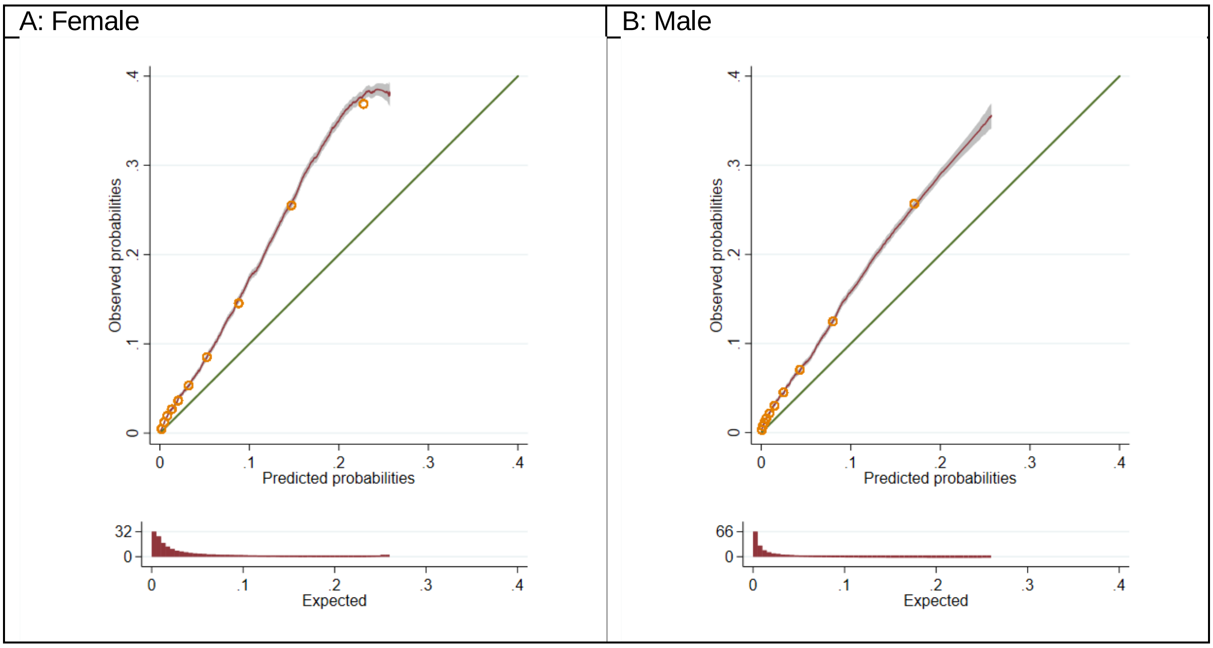

Table S2.2: Predictive performance statistics by sex

	Female	Male
**N**	1,959,472	1,845,850
**Falls events (%)**	134,945 (6.9%)	72,011 (3.9%)
**Observed/Expected**		
Pooled effect size (95% CI)	1.817 (1.790 to 1.842)	1.839 (1.804 to 1.874)
Prediction interval	1.259 to 2.612	1.139 to 2.974
Tau^2	0.034 (0.030 to 0.038)	0.060 (0.054 0.067)
**C statistic**		
Pooled effect size (95% CI)	0.843 (0.842 to 0.845)	0.829 (0.828 to 0.831)
Prediction interval	0.804 to 0.875	0.786 to 0.865
Tau^2	0.019 (0.016 to 0.022)	0.020 (0.017 to 0.024)
**D statistic**		
Pooled effect size (95% CI)	1.498 (1.381 to 1.616)	1.669 (1.538 to 1.800)
Prediction interval	1.38 to 1.62	1.54 to 1.80
Tau^2	0.000 (0.000 to 0.014)	0.000 (0.000 to 0.018)
**Royston and Sauerbrei's R_D^2**		
Range	12.2 to 66.4	10.9 to 93.6
Median (IQR)	36.3 (32.5 to 40.8)	41.4 (37.2 to 46.2)
Mean (SD)	37.2 (7.8)	42.3 (8.1)

Figure S2.4: Decision curve analysis by sex

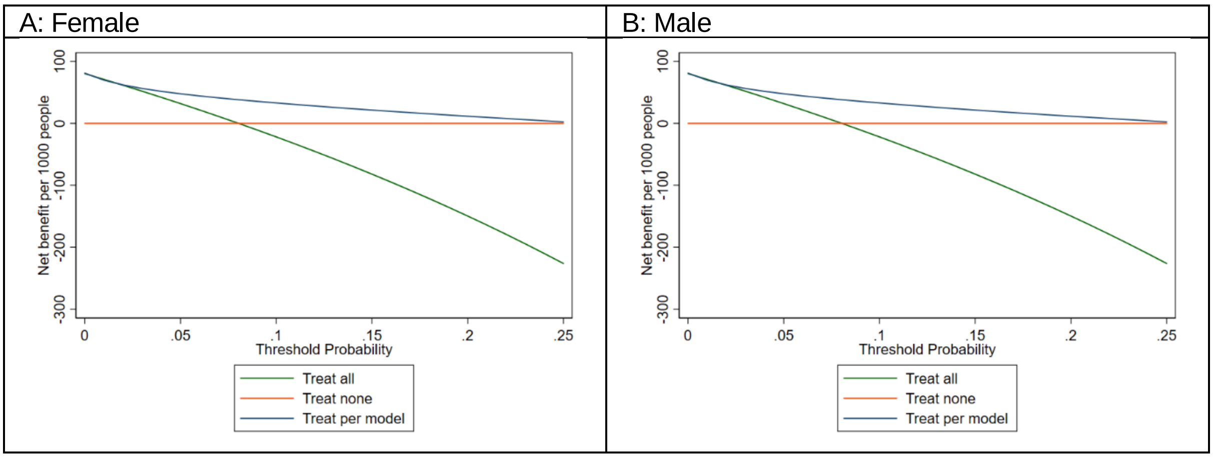

Model performance by ethnicity

Figure S2.5: Calibration plots by ethnicity

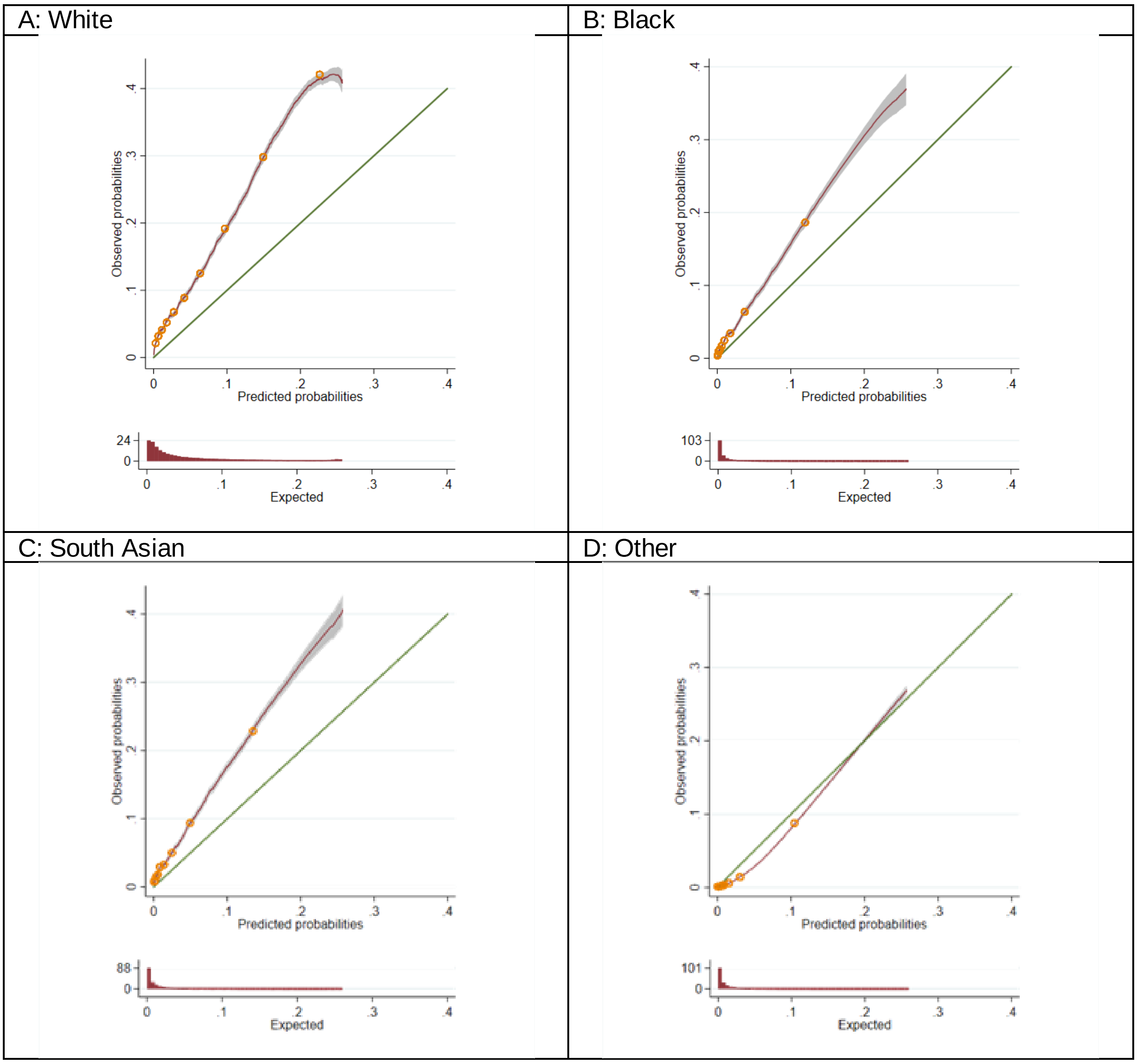

Table S2.3: Predictive performance statistics by ethnicity group

	White	Black	South Asian	Other
**N**	2,041,469	115,276	94,483	832,614
**Falls events (%)**	194,311 (9.5%)	2,239 (1.9%)	2,449 (2.6%)	3,442 (0.4%)
**Observed/Expected**				
Pooled effect size (95% CI)	2.140 (2.106 to 2.175)	2.795 (2.535 to 3.086)	2.606 (2.408 to 2.821)	0.433 (0.396 to 0.472)
Prediction interval	1.433 to 3.19	0.527 to 14.88	0.577 to 11.71	0.056 to 3.353
Tau^2 (95% CI)	0.042 (0.038 to 0.047)	0.718 (0.611 to 0.850)	0.584 (0.505 to 0.679)	1.081 (0.962 to 1.220)
**C statistic**				
Pooled effect size (95% CI)	0.796 (0.793 to 0.798)	0.812 (0.803 to 0.821)	0.815 (0.806 to 0.824)	0.834 (0.830 to 0.839)
Prediction interval	0.735 to 0.846	0.729 to 0.874	0.721 to 0.883	0.752 to 0.894
Tau^2 (95% CI)	0.029 (0.026 to 0.034	0.057 (0.026 to 0.102)	0.072 (0.035 to 0.124)	0.067 (0.051 to 0.086)
**D statistic**				
Pooled effect size (95% CI)	1.291 (1.189 to 1.393)	0.751 (0.608 to 0.895)	0.85 (0.709 to 0.992)	1.115 (0.981 to 1.25)
Prediction interval	1.19 to 1.39	-0.04 to 1.55	-0.03 to 1.73	0.11 to 2.12
Tau^2 (95% CI)	0.000 (0.000 to 0.012)	0.157 (0.068 to 0.312)	0.194 (0.094 to 0.352)	0.255 (0.146 to 0.412)
**Royston and Sauerbrei's R_D^2**				
Range	0.0 to 100.0	0.0 to 100.0	0.0 to 100.0	0.0 to 100.0
Median (IQR)	31.5 (26.8 to 36.1)	51.4 (29.1 to 72.4)	53.3 (35.0 to 74.5)	58.7 (40.7 to 71.2)
Mean (SD)	31.8 (8.6)	51.1 (30.2)	53.7 (28.9)	54.3 (0.24.2)

Figure S2.6: Decision curve analysis by ethnicity group

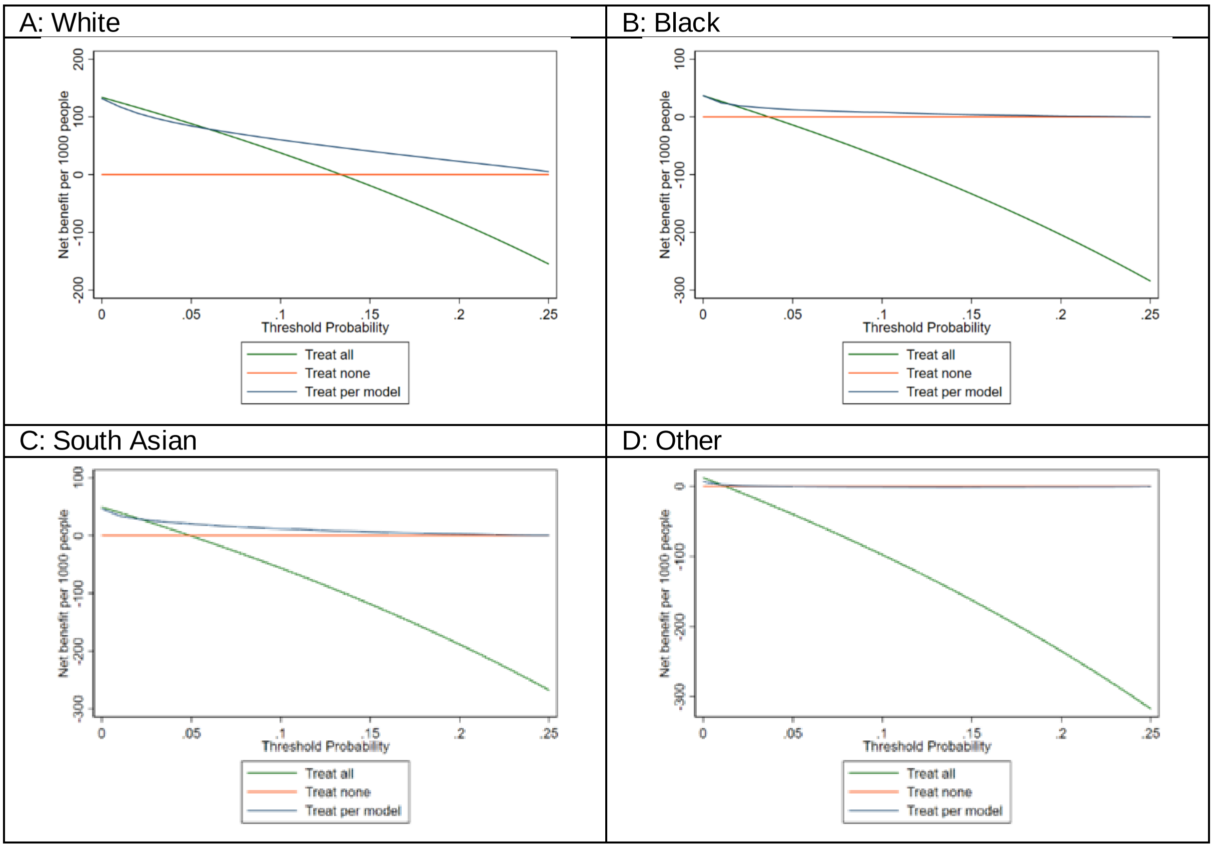

3 Further supplementary figures

Figure S3.1: Calibration curves for the apparent performance of the original model in CPRD GOLD, and the calibration on external validation in CPRD Aurum.

	**Calibration in GOLD (development data)**	**Calibration in Aurum (external validation)**
**1 year**	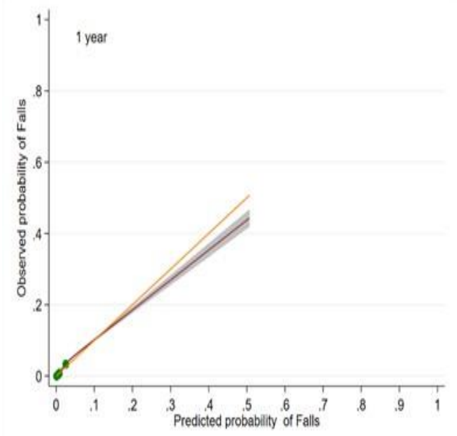	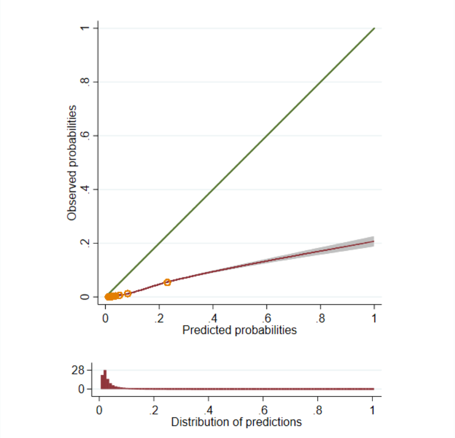
**5 years**	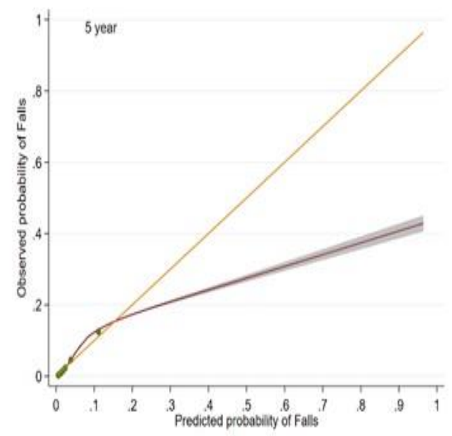	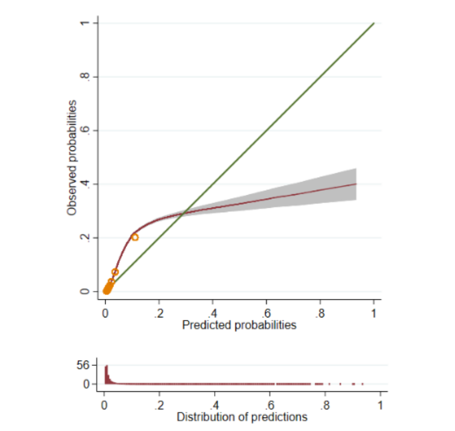
**10 years**	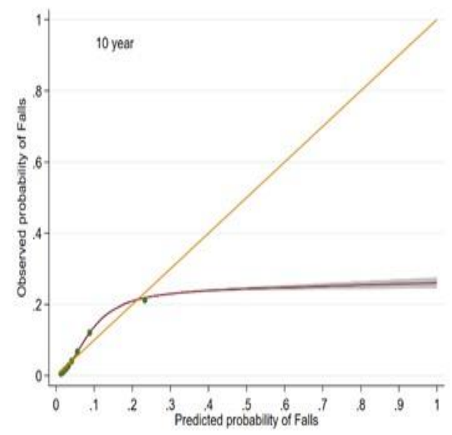	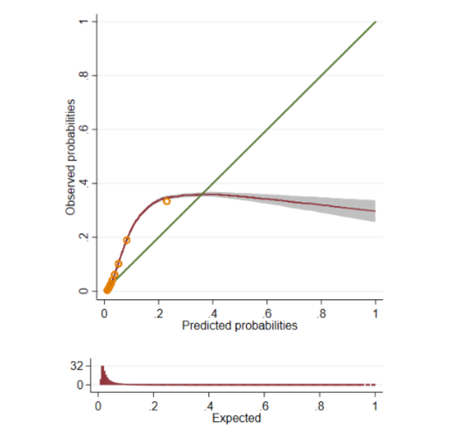

Figure S3.2: Performance variability of the original model on external validation across GP practices, with panels (A) Observed/Expected, (B) R_D^2, (C) D-statistic, and (D) C-statistic

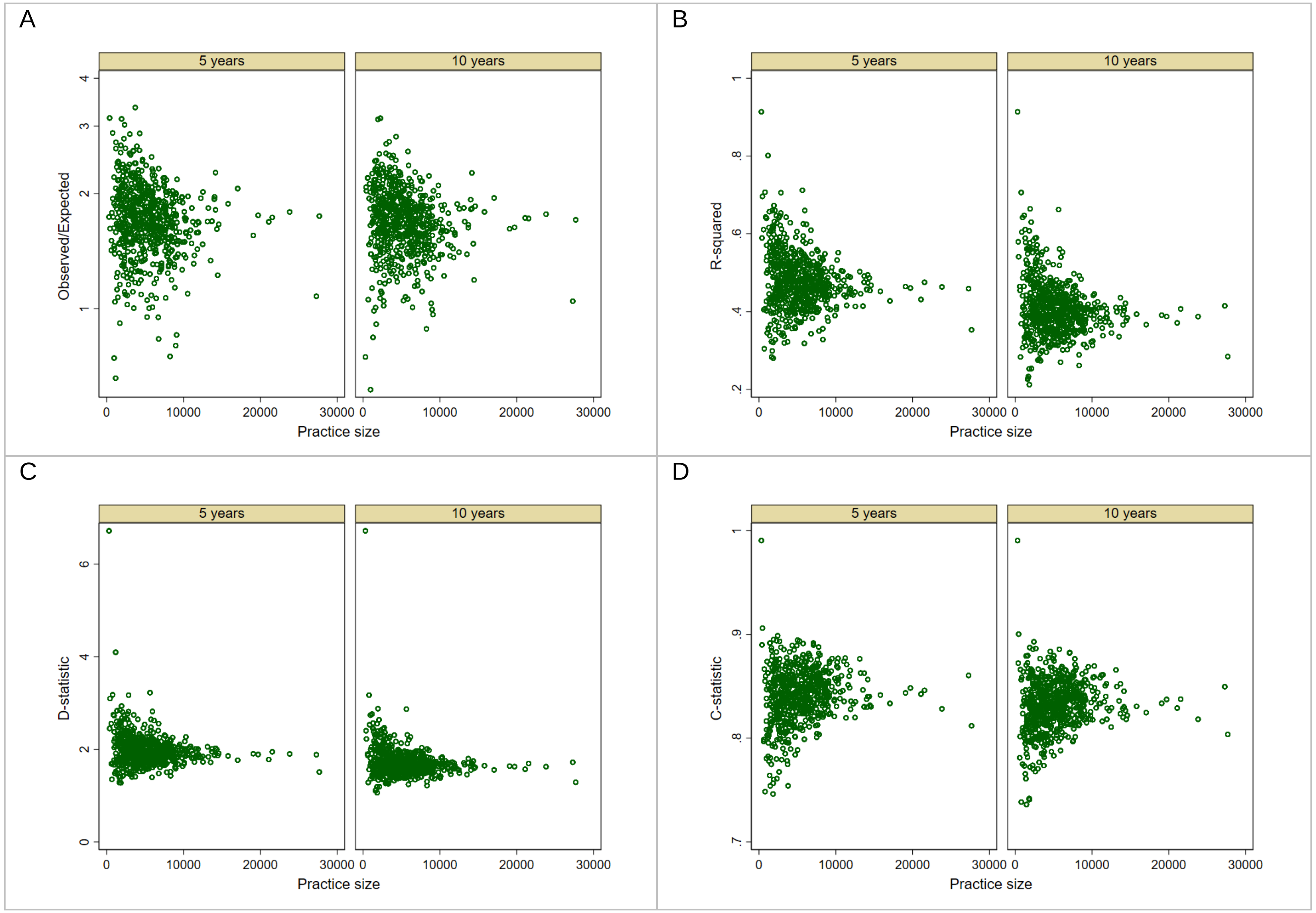

Figure S3.3: Falls incidence by practice at 5 and 10 years

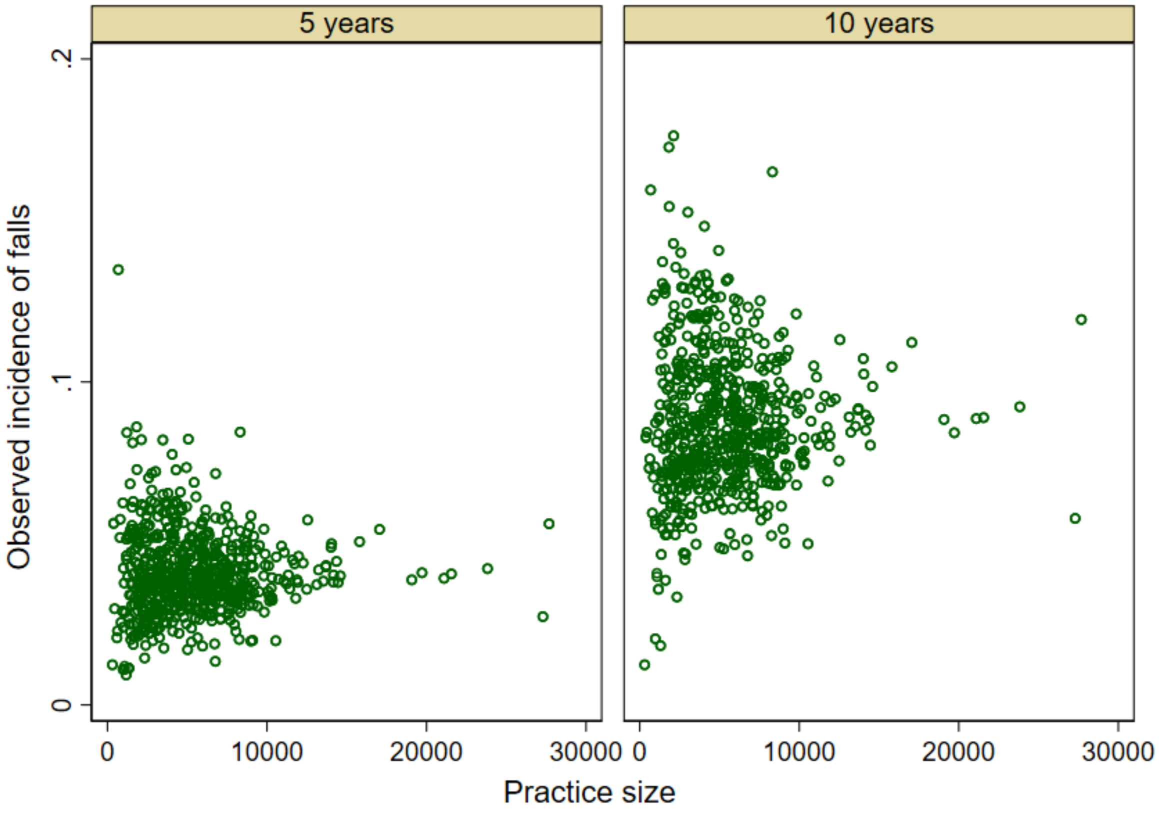

Figure S3.4: Distribution of linear predictor, grouped by outcome type

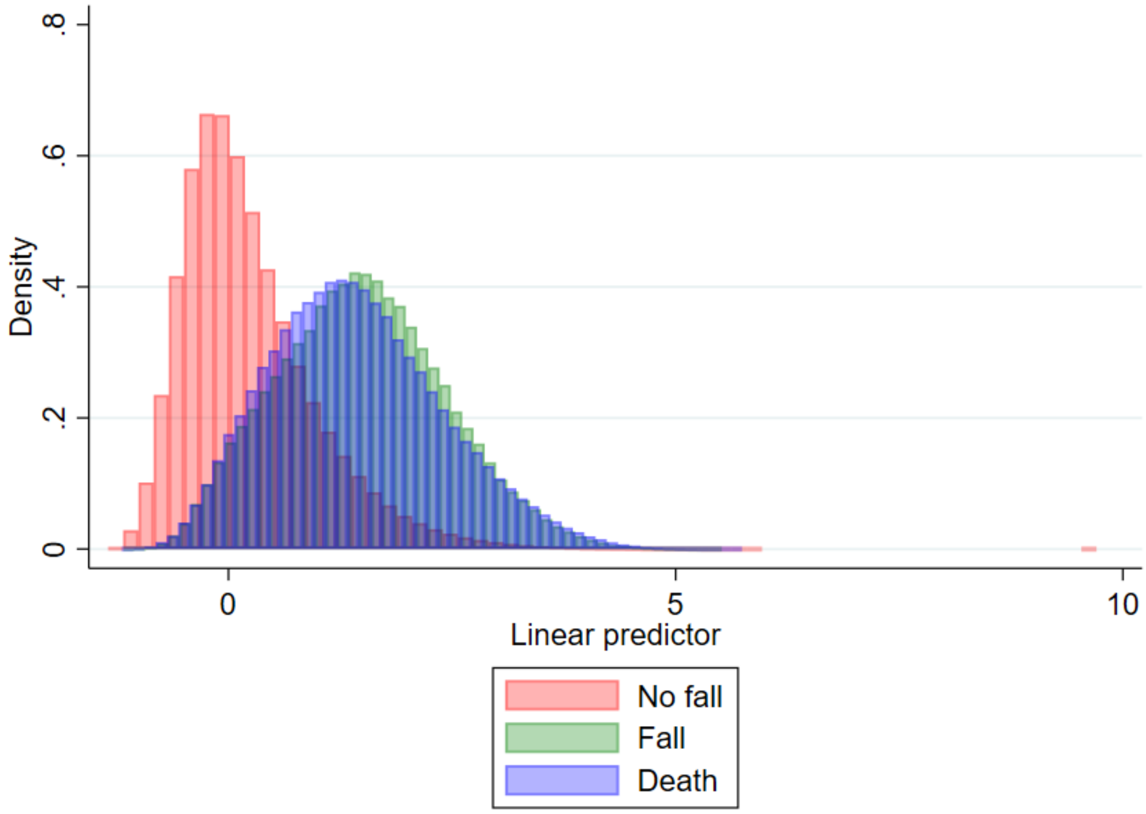

4 Further supplementary tables

Table S4.1: ICD9/10 codes for Fall

Code	Description
W01	Fall on same level from slipping, tripping and stumbling
W05	Fall involving wheelchair
W06	Fall involving bed
W07	Fall involving chair
W08	Fall involving other furniture
W10	Fall on and from stairs and steps
W18	Other fall on same level
W19	Unspecified fall
R29.6	Tendency to fall, not elsewhere classified
E880	fall on stairs or steps*
E880.0	fall on escalator
E880.1	fall on sidewalk curb
E880.9	fall on stair/step nec
E884.2	fall from chair
E884.3	fall from wheelchair
E884.4	fall from bed
E884.5	fall from furniture nec
E884.6	fall from commode
E885	fall on level-tripping*
E885.9	fall from slipping nec
E886.9	fall on level nec/nos
E888	fall nec & nos*
E888.8	fall nec
E888.9	fall nos

Table S4.2: Predictors included in the model development

**Predictor**	**Description**	**Categorisation**	**Final model**
Age	Estimated in years	Continuous	Included
Sex	Male or female	Binary	Included
Ethnicity	White, Black, South Asian, Other	Categorical	Included
Socioeconomic deprivation	Based on indices of multiple deprivation (IMD) score, estimated from patient post code	Categorical	Included
Systolic blood pressure	Based on most recent clinic reading, in mm Hg	Continuous	**Excluded**
Diastolic blood pressure	Based on most recent clinic reading, in mm Hg	Continuous	**Excluded**
Body mass index	Based on most recent clinic readings of height and weight, categorised as non-drinker, trivial drinker, light drinker, moderate drinker, heavy drinker, unknown amount	Categorical	**Excluded**
Total cholesterol	Based on most recent clinic reading, in mmol/L	Continuous	Included
Smoking	Non-smoker, ex-smoker, current smoker	Categorical	Included
Alcohol intake	Based on most recent clinic readings of height and weight, categorised as underweight, normal, overweight, obese, morbidly obese	Categorical	Included
Falls	Previous history	Binary	Included
Memory issues	Previous history (based on definition used in electronic frailty index)	Binary	Included
Mobility issues	Previous history (based on definition used in electronic frailty index)	Binary	Included
Stroke	Previous history	Binary	Included
Multiple sclerosis	Previous history	Binary	Included
Activity limitation	Previous history (based on definition used in electronic frailty index)	Binary	**Excluded**
Syncope	Previous history	Binary	**Excluded**
Cataract	Previous history	Binary	**Excluded**
Frailty	Based on the electronic frailty index	Continuous	Included
Antihypertensives entered into the model as separate drug classes:<br>ACE inhibitors,<br>Angiotensin II receptor blockers,<br>Alpha blockers,<br>Beta blockers,<br>Calcium channel blockers,<br>Diuretics<br>Other antihypertensives	Acebutolol hydrochloride	Binary	Included
	Aliskiren hemifumarate		
	Ambrisentan		
	Amlodipine		
	Atenolol		
	Azilsartan medoxomil		
	Bendroflumethiazide		
	Bisoprolol Fumarate		
	Bosentan monohydrate		

	Candesartan cilexetil		
	Captopril		
	Carteolol Hydrochloride		
	Carvedilol		
	Celiprolol hydrochloride		
	Chlortalidone		
	Clonidine		
	Diazoxide		
	Diltiazem Hydrochloride		
	Doxazosin mesilate		
	Enalapril Maleate		
	Eprosartan mesilate		
	Esmolol Hydrochloride		
	Felodipine		
	Fosinopril sodium		
	Hydralazine Hydrochloride		
	Hydrochlorothiazide		
	Iloprost trometamol		
	Imidapril hydrochloride		
	Indapamide		
	Indoramin Hydrochloride		
	Irbesartan		
	Isradipine		
	Labetalol hydrochloride		
	Lacidipine		
	Lercanidipine hydrochloride		
	Lidoflazine		
	Lisinopril		
	Losartan potassium		
	Macitentan		
	Meprobamate/Bendroflumethiazide		
	Methyldopa Anhydrous		
	Methyldopate Hydrochloride		
	Metolazone		
	Metoprolol		
	Metoprolol Tartrate		
	Mibefradil		
	Minoxidil		
	Moexipril hydrochloride		
	Moxonidine		
	Nadolol		
	Nebivolol hydrochloride		
	Nicardipine hydrochloride		
	Nifedipine		

	Nimodipine		
	Nisoldipine		
	Olmesartan medoxomil		
	Oxprenolol Hydrochloride		
	Oxprenolol Hydrochloride/Cyclopenthiazide		
	Perhexiline Maleate		
	Perindopril Erbumine		
	Perindopril arginine		
	Perindopril tosilate		
	Phentolamine mesilate		
	Pindolol		
	Pindolol/Clopamide		
	Potassium Chloride/Chlortalidone		
	Practolol		
	Prazosin Hydrochloride		
	Prenylamine Lactate		
	Propranolol Hydrochloride		
	Quinapril Hydrochloride		
	Quinapril hydrochloride		
	Ramipril		
	Reserpine/Bendroflumethiazide		
	Reserpine/Hydrochlorothiazide		
	Riociguat		
	Sacubitril/Valsartan		
	Sildenafil Citrate		
	Sodium nitroprusside dihydrate		
	Sotalol Hydrochloride/Hydrochlorothiazide		
	Tadalafil		
	Telmisartan		
	Terazosin hydrochloride		
	Terazosine		
	Timolol maleate		
	Timolol maleate/Amiloride hydrochlori..		
	Trandolapril		
	Triamterene/Chlortalidone		
	Valsartan		
	Verapamil hydrochloride		
	Xipamide		
**Anticholinergics**	Amantadine hydrochloride	Binary	Included
	Amitriptyline hydrochloride/Perphenazine		
	Aspirin/Methocarbamol		
	Atropine/Isoprenaline Hydrochloride		

	Atropine/Papaverine Hydrochloride/Adrenaline		
	Atropine/Papaverine Hydrochloride/Adrenaline Acid Tartrate		
	Atropine/Papaverine Hydrochloride/Adrenaline Hydrochloride/Benzocaine		
	Atropine sulfate		
	Benzatropine mesilate		
	Brompheniramine maleate		
	Chlorpromazine embonate		
	Chlorpromazine Hydrochloride		
	Cinnarizine/Dimenhydrinate		
	Clozapine		
	Darifenacin hydrobromide		
	Dimenhydrinate		
	Diphenoxylate Hydrochloride/Atropine Sulphate		
	Fesoterodine fumarate		
	Flavoxate Hydrochloride		
	Guaifenesin/Pseudoephedrine hydrochloride/Brompheniramine maleate		
	Homatropine hydrobromide		
	Isopropamide Iodide/Trifluoperazine Hydrochloride		
	Methocarbamol		
	Nefopam hydrochloride		
	Olanzapine		
	Orphenadrine hydrochloride		
	Oxcarbazepine		
	Oxybutynin		
	Paracetamol/Orphenadrine Hydrochloride		
	Paroxetine hydrochloride		
	Perphenazine		
	Propantheline bromide		
	Propiverine hydrochloride		
	Pseudoephedrine Hydrochloride/Brompheniramine Maleate		
	Quetiapine fumarate		
	Solifenacin succinate		
	Thioridazine		
	Tolterodine tartrate		
	Trifluoperazine hydrochloride		
	Trospium chloride		
**Antidepressants**	Amitriptyline hydrochloride	Binary	Included

	Buspirone hydrochloride		
	Clomipramine hydrochloride		
	Dosulepin hydrochloride		
	Doxepin hydrochloride		
	Duloxetine hydrochloride		
	Escitalopram oxalate		
	Imipramine hydrochloride		
	Isocarboxazid		
	Lofepramine hydrochloride		
	Mianserin hydrochloride		
	Mirtazapine		
	Moclobemide		
	Nortriptyline hydrochloride		
	Paroxetine hydrochloride		
	Phenelzine sulfate		
	Sertraline		
	Sertraline hydrochloride		
	Tranylcypromine sulfate		
	Trazodone hydrochloride		
	Trifluoperazine Hydrochloride/Tranylcypromine Sulphate		
	Trimipramine maleate		
	Venlafaxine hydrochloride		
	Vortioxetine hydrobromide		
	Alprazolam		
**Hypnotics, anxiolytics**	Buspirone hydrochloride	Binary	Included
	Chlordiazepoxide Hydrochloride		
	Clobazam		
	Clomethiazole		
	Diazepam		
	Flunitrazepam		
	Flurazepam hydrochloride		
	Hydroxyzine hydrochloride		
	Loprazolam mesilate		
	Lorazepam		
	Lormetazepam		
	Meprobamate		
	Nitrazepam		
	Oxazepam		
	Promethazine hydrochloride		
	Temazepam		
	Thiopental sodium		
	Zolpidem tartrate		
	Zopiclone		

	Alfentanil Hydrochloride		
**Opioids**	Buprenorphine	Binary	Included
	Codeine phosphate		
	Cyclizine Tartrate/Morphine Tartrate		
	Diamorphine hydrochloride		
	Dihydrocodeine Tartrate		
	Ethylmorphine hydrochloride		
	Fentanyl		
	Meptazinol hydrochloride		
	Methadone hydrochloride		
	Morphine Anhydrous		
	Morphine hydrochloride		
	Morphine Sulphate		
	Naloxone hydrochloride		
	Oxycodone hydrochloride		
	Pentazocine hydrochloride		
	Pentazocine lactate		
	Pethidine hydrochloride		
	Remifentanyl hydrochloride		
	Tapentadol hydrochloride		
	Tramadol hydrochloride		

Codelists for each predictor can be found at:
https://github.com/jamessheppard48/STRATIFY-BP/tree/STRATIFY-Falls

Table S4.3: TRIPOD checklist

Section/Topic			Checklist Item	Page
**Title and abstract**				
Title	1	D;V	Identify the study as developing and/or validating a multivariable prediction model, the target population, and the outcome to be predicted.	P1
Abstract	2	D;V	Provide a summary of objectives, study design, setting, participants, sample size, predictors, outcome, statistical analysis, results, and conclusions.	P4/5
**Introduction**				
Background and objectives	a	D;V	Explain the medical context (including whether diagnostic or prognostic) and rationale for developing or validating the multivariable prediction model, including references to existing models.	P6
	b	D;V	Specify the objectives, including whether the study describes the development or validation of the model or both.	P7
**Methods**				
Source of data	a	D;V	Describe the study design or source of data (e.g., randomized trial, cohort, or registry data), separately for the development and validation data sets, if applicable.	P8
	b	D;V	Specify the key study dates, including start of accrual; end of accrual; and, if applicable, end of follow-up.	P8
Participants	a	D;V	Specify key elements of the study setting (e.g., primary care, secondary care, general population) including number and location of centres.	P8
	b	D;V	Describe eligibility criteria for participants.	P8
	c	D;V	Give details of treatments received, if relevant.	NA
Outcome	a	D;V	Clearly define the outcome that is predicted by the prediction model, including how and when assessed.	P9
	b	D;V	Report any actions to blind assessment of the outcome to be predicted.	NA
Predictors	a	D;V	Clearly define all predictors used in developing or validating the multivariable prediction model, including how and when they were measured.	P9/10
	b	D;V	Report any actions to blind assessment of predictors for the outcome and other predictors.	NA
Sample size	3	D;V	Explain how the study size was arrived at.	10+Supp1.1
Missing data	9	D;V	Describe how missing data were handled (e.g., complete-case analysis, single imputation, multiple imputation) with details of any imputation method.	P10/11
Statistical analysis methods	0a	D	Describe how predictors were handled in the analyses.	P11
	0b	D	Specify type of model, all model-building procedures (including any predictor selection), and method for internal validation.	P11/12
	0c	V	For validation, describe how the predictions were calculated.	P12/B1
	0d	D;V	Specify all measures used to assess model performance and, if relevant, to compare multiple models.	P12/13
	0e	V	Describe any model updating (e.g., recalibration) arising from the validation, if done.	P12
Risk groups	1	D;V	Provide details on how risk groups were created, if done.	NA
Development vs. validation	2	V	For validation, identify any differences from the development data in setting, eligibility criteria, outcome, and predictors.	P8
**Results**				
Participants	3a	D;V	Describe the flow of participants through the study, including the number of participants with and without the outcome and, if applicable, a summary of the follow-up time. A diagram may be helpful.	P15
	3b	D;V	Describe the characteristics of the participants (basic demographics, clinical features, available predictors), including the number of participants with missing data for predictors and outcome.	T1/ P15
	3c	V	For validation, show a comparison with the development data of the distribution of important variables (demographics, predictors and outcome).	T1/P15
Model development	4a	D	Specify the number of participants and outcome events in each analysis.	P15
	4b	D	If done, report the unadjusted association between each candidate predictor and outcome.	NA
Model specification	5a	D	Present the full prediction model to allow predictions for individuals (i.e., all regression coefficients, and model intercept or baseline survival at a given time point).	T2
	5b	D	Explain how to the use the prediction model.	B1
Model performance	6	D;V	Report performance measures (with CIs) for the prediction model.	T3/P16/17
Model-updating	7	V	If done, report the results from any model updating (i.e., model specification, model performance).	NA
**Discussion**				
Limitations	8	D;V	Discuss any limitations of the study (such as nonrepresentative sample, few events per predictor, missing data).	P18/19/20
Interpretation	9a	V	For validation, discuss the results with reference to performance in the development data, and any other validation data.	P20

	9b	D;V	Give an overall interpretation of the results, considering objectives, limitations, results from similar studies, and other relevant evidence.	P20/21
Implications	0	D;V	Discuss the potential clinical use of the model and implications for future research.	P21/22
**Other information**				
Supplementary information	1	D;V	Provide information about the availability of supplementary resources, such as study protocol, Web calculator, and data sets.	B1/P25/P8
Funding	2	D;V	Give the source of funding and the role of the funders for the present study.	P23/24

5 CPRD Independent Scientific Advisory Committee (ISAC) (protocol number 19_042) - PART 2: PROTOCOL INFORMATION

**Applicants must complete all sections listed below**<br>**Sections which do not apply should be completed as '*Not Applicable*' and justification provided**
**A.** Study Title (Max. 255 characters)<br><br>Understanding the harms of antihypertensives, statins and antiplatelets for prevention of cardiovascular disease: A prognostic modelling and casual inference study
**B.** Lay Summary (Max. 250 words)<br><br>People are living for longer, with more long-term physical and mental conditions which worsen their health. One example is high blood pressure, where people can take 3-4 drugs to prevent stroke. However, dozens of people have to be treated for at least a year to prevent a stroke in one person. This is because these drugs only reduce the possibility of stroke, they do not remove it altogether. Some of these patients may be prone to side effects such as falls and kidney problems which may be more common than any benefits.<br><br>This proposal aims to use information from the medical records from hundreds of thousands of patients to establish the link between drugs used to prevent heart attack and stroke and side effects. We will focus on drugs that lower blood pressure (known as antihypertensives), lower cholesterol (known as statins) or prevent blood clots (known as antiplatelets). This information will be used to develop a calculator which predicts a person's risk of experiencing side effects. This calculator will form part of a support tool which will help patients and doctors make better informed decisions about starting or continuing drugs.

C. Technical Summary (Max. 300 words)

Background

The population is ageing and consequently, the number of people living with age-related chronic conditions is increasing. Polypharmacy (five or more prescribed medications) is common in older people and is associated with an increased risk of adverse drug reactions. Preventative medications, such as those used to manage blood pressure and cholesterol, are common in polypharmacy and often require large numbers of people to be treated to prevent a small number of cardiovascular disease (CVD) events. This leaves many individuals on drugs of little benefit, some of whom may be susceptible to side effects such as falls, kidney problems and muscle pain.

Aims

This proposal aims to quantify the harms of cardiovascular prevention medication, and the characteristics of those people most likely to suffer them. This research is one part of a larger research programme to develop a clinical decision tool which estimates an individual's likelihood of benefiting or suffering harm from treatment.

Methods

Aim 1: Derive prognostic models for an individual's risk of adverse events associated with cardiovascular prevention treatment (antihypertensives, statins and antiplatelets) using data from the CPRD GOLD. Adverse event outcomes will include falls (antihypertensives), myopathy (statins) and bleeding (antiplatelets).

Aim 2: Externally validate each model using data from the CPRD Aurum.

Aim 3: Use causal inference methods (propensity score matching, instrumental variable analysis) to examine whether modification of treatment could have an important impact on the risk of adverse events.

D. Outcomes to be measured

Primary outcomes - Falls; Myopathy/muscle pain; Intracerebral haemorrhage

Secondary outcomes – Fracture; Syncope; Hypotension (symptomatic); Acute kidney injury; Electrolyte abnormalities; Gout; Diabetes mellitus; Intracerebral haemorrhage; Cataract; Liver dysfunction; Dementia; Memory problems (including dementia); Gastrointestinal haemorrhage; Dyspnoea; Any adverse event

Specific outcomes will be examined in relation to exposure to either antihypertensives, statins or antiplatelets (details given in section N).

E. Objectives, Specific Aims and Rationale

Objective

The overarching objective of this study is to better understand the harms of preventative treatments (antihypertensives, statins and antiplatelets) by quantifying an individual's baseline risk of harm and the modifying effect of treatment.

Specific aims

Aim 1: Derive prognostic models for an individual's risk of adverse events associated with cardiovascular prevention treatment (antihypertensives, statins and antiplatelets). Adverse event outcomes will include falls (antihypertensives), myopathy (statins) and bleeding (antiplatelets).

Aim 2: Externally validate each model.

Aim 3: Use causal inference methods (propensity score matching, instrumental variable analysis) to examine whether modification of treatment could have an important impact on the risk of adverse events.

Rationale

The proposed work will develop and validate new prediction tools for an individual's risk of harms from cardiovascular prevention medication. This information will be combined with existing tools for the benefits of treatment and be used to form a new strategy that better targets preventative therapy at those with the most to gain. By empowering patients and clinicians to better understand the risks of preventative medications, this work will promote a more patient-centred, shared-decision making approach to cardiovascular disease prevention in primary care.

F. Study Background

Cardiovascular disease (CVD) is the leading cause of mortality worldwide.[1] As a result, much healthcare

resource is assigned to preventing CVD through modification of risk factors, such as raised blood pressure and/or cholesterol.[2] This can be achieved through prescription of medications such as antihypertensives, statins and antiplatelets, which have been shown to be effective in reducing the risk of cardiovascular disease.[3-5] These medications are often started when patients are at low-to-moderate risk and continued for many years, despite a low likelihood of benefit.[6] Some patients who take these medications may suffer side effects such as falls, acute kidney injury (AKI), muscle pain and bleeding which can significantly reduce an individual's quality of life, particularly those who are old and frail.[7-9] Despite changes in an individual's risk/benefit profile, clinicians are often reluctant to stop prescribing them.[10]

The association between cardiovascular preventative therapy and adverse events is not fully understood. This is due in part, to the completeness of reporting of side effects and adverse events in previous trials.[11 12] Observational studies do show an association between treatment and adverse outcomes such as falls,[9 13 14] AKI,[15] myopathy,[16] diabetes,[17] and intracranial bleeding,[18] but are limited in some cases due to small sample sizes and bias caused by unmeasured confounding. Observational data can however be useful since it allows multi-morbid populations to be examined, which are more representative of the general population than those enrolled into clinical trials.[19]

Predicting the benefits and harms of treatment

A structured approach to weighing up the risks and benefits of treatment is common in atrial fibrillation, where combining risk scores from the CHA_2DS_2-VASc tool[20] and HAS-BLED tool[21] allows one to compare an individual's risk of stroke with their risk of a bleed. There are many other risk prediction tools which can be used to identify individuals who may benefit from CVD prevention treatment (i.e. are at high risk of CVD),[22-28] but very few which can be used to identify those at risk of adverse events in the community. Existing tools show moderate discrimination and focus on patients with specific conditions and/or use in an acute hospital setting.[29-34] None have been externally validated.[35-37] Recent studies have described prediction models for the benefits and harms of *intensive* blood pressure lowering treatment,[38 39] but these were derived exclusively from patients in the SPRINT trial,[7] limiting their generalisability.

Stratifying treatments for prevention of CVD is important for UK and international health. Such an approach could facilitate shared decision making between patients and doctors and allow better targeting of treatments in settings where resources and access to drugs may be limited.

How this project fits in

This is one project in a larger work programme aiming to personalise preventative treatments for cardiovascular disease. In this project, we will quantify risks due to cardiovascular preventive treatments. In other projects, including systematic reviews, we will quantify and combine estimates of the benefits and harms of preventive treatments. This will enable us to develop a clinical decision tool to assist patients and their clinicians in understanding the relative benefits and harms of proposed treatment changes.

**G.** Study Type<br><br>Hypothesis testing
**H.** Study Design<br><br>Longitudinal cohort study
**I.** Feasibility counts<br><br>There are approximately 5.5 million patients in England, aged 40+ and with up to standard registration for a period between 1/1/1998 to 31/12/2017 in the CPRD GOLD. Restricting to English practices (linked to HES and ONS) and only including patients fulfilling the eligibility criteria listed below, the expected population is approximately 3.3 million and out of these we expect 216,000 fall events, 230,000 myopathy/muscle pain events and 1,200 intracranial haemorrhage events.
**J.** Sample size considerations<br><br>We have based our sample size calculation on the least common of the primary outcomes listed above (intracranial haemorrhage), and used the method developed by Riley *et al.,*[40] to minimise the potential for overfitting and ensure precise estimates of key parameters. A sample size of approximately 80,000 patients is required for the development of a clinical prediction rule for this outcome, assuming an event rate of 24.6 per 100,000 person years,[41] a median follow up of 7 years,[42] a conservative estimate of Nagelkerke's R^2 statistic of 0.15 and a maximum number of 40 parameters per model.<br><br>Feasibility counts showed that this sample size is adequate for the development of the prediction rules. Since Intracranial bleeding is the rarest of the primary outcomes to be studied, the sample size will be sufficient for all other outcomes of interest. For validation, Vergouwe *et al.,*[43] consider at least 100 events per sample population to be "substantial" and we will comfortably exceed this in our validation cohorts for each outcome of interest.<br><br>For analyses using causal inference methods (propensity score matching and instrumental variable analysis), assuming clinically significant increased rate of each adverse event with treatment of 10%, and an event rate of at least 0.5% per year in the non-exposed group, approximately 88,380 patients (44,190 in each group) and 4,634 events will be required to accurately define the relationship between preventative treatment and adverse events, with 90% power and an alpha of 0.05.

K. Planned use of linked data (if applicable):

Data linkage to the ONS and Basic HES are required to define the primary and secondary outcomes in the study. The ONS mortality register will be used to define any outcomes which result in death, and also censor follow-up at death. Specific linkages required will include data and ICD-10 coded cause of death (see attached code lists). Linkages to Basic Inpatient HES will be combined with data from the ONS to define all other outcomes in the study. HES data will also be used to define patients' eligibility for the study (e.g. previous CVD), and define the study population (e.g. ethnicity where unavailable in Primary Care records). Data required from Basic Inpatient HES will include primary diagnosis, secondary diagnosis, patient characteristics (e.g. sex, ethnicity), date of admission and date of discharge. All deaths and hospital admissions occurring after a patient's index date will be included. A linkage to the Index of Multiple Deprivation is required to acquire patient level quintiles of multiple deprivation, to better define the sample population and use as a covariate in the prognostic modelling.

L. Definition of the Study population

Individual patient data will be extracted from the medical records of all patients registered at linked general practices contributing to the CPRD GOLD and Aurum in England.

Patients will be included if they meet the following criteria:

- Patients over the age of 40 years
- Registered to a CPRD 'up-to-standard' practice
- Records available after the study start date (01/01/1998)
- Sufficient data to define the index date (see table below)

Patients will be excluded if they meet the following criteria:

- Previous prescription of the exposure variable (antihypertensive, statin or antiplatelet)
- Blood pressure >180 mm Hg or total cholesterol >7.5 mmol/L (in patients with high blood pressure or cholesterol, treatment is indicated regardless of risk)

Study entry criteria – Patients will enter the cohort on the index date, defined as:

**Exposure**	Antihypertensive therapy	Statin therapy	Antiplatelet therapy
**Index date definition**	12 months after the first systolic blood pressure reading ≥130 mm Hg	12 months after the first coded or calculated CVD risk of ≥5%	3 months after first CVD event

Study exit criteria

- Last date at which the most recent linked data are available from the CPRD (study end date, July 2018)
- Date of the most recent data upload from the practice to which a given patient is registered
- Date at which a given patient transfers out of a registered CPRD practice
- Date of death or specific outcome of interest

M. Selection of comparison group(s) or controls

Aim 1: Not applicable – single cohort in CPRD GOLD.

Aim 2: Not applicable – single cohort in CPRD AURUM.

Aim 3 will use propensity score matching and instrumental variable analysis – see Methods section below.

N. Exposures, Outcomes and Covariates

This study will examine the overall risk of harm from three types of cardiovascular medication: antihypertensives, statins and antiplatelets. 'Harm' will be defined by the outcomes listed in the table below, which will be examined both individually and as a composite outcome. The association between treatment and a positive and negative control outcome will also be examined to check the validity of the treatment effect estimates. Positive controls are outcomes know to be affected by treatment (e.g. cardiovascular disease) and negative outcomes are those known not to be affected by treatment.

Variable	Antihypertensive therapy	Statin therapy	Antiplatelet therapy
Exposure	ACE inhibitors<br>Angiotensin II receptor antagonists<br>Calcium channel blockers<br>Thiazides and thiazide-like diuretics<br>Beta blockers<br>Alpha blockers<br>Centrally acting antihypertensives<br>Vasodilators<br>Renin inhibitors	Statins	Aspirin<br>Clopidogrel<br>Dipyridamole<br>Ticagrelor<br>Prasugrel
Outcome (hospital admission or death due to)	Falls<br>Fracture<br>Syncope<br>Hypotension (symptomatic)<br>Acute kidney injury<br>Electrolyte abnormalities*<br>Gout<br>Any adverse event	Myopathy/muscle pain<br>Diabetes mellitus<br>Intracerebral haemorrhage<br>Cataract<br>Liver dysfunction<br>Dementia<br>Memory problems (including dementia)<br>Any adverse event	Intracerebral haemorrhage<br>Gastrointestinal haemorrhage<br>Dyspnoea<br>Any adverse event
Covariates (predictors of outcome and/or treatment)	Age, sex, BMI, blood pressure, cholesterol, HbA1c, other prescribed medications, co-morbidities, social deprivation, previous adverse events, frailty and proximity to death.		

*Defined as any (hyponatraemia, hypokalaemia, hyperkalaemia, hypomagnesaemia, hypophosphataemia hypercalcaemia or hyperuricemia)

All outcomes will be defined according to diagnostic and/or symptom codes, unless otherwise stated. Further sensitivity analyses will explore the impact of our definition of myopathy and muscle pain, focussing on formal diagnostic codes for myopathy only.

Our PPI engagement suggests some patients are concerned about side effects which will result in having to go to hospital, whilst others are more concerned about chronic conditions which affect their daily quality of life. The primary outcomes of this study will therefore be hospital attendance, defined according to ONS and Basic HES datasets. Sensitivity analyses will explore outcomes defined more broadly using primary care codes as well.

All published outputs from this work will be based on the outcomes as specified in this application. Further work with our patient and public involvement representatives will explore definitions of these outcomes and how they should be incorporated into the final calculators developed as part of the wider programme of work related to this project.

Each model will be derived pragmatically, using coded data routinely available in a primary care setting. Potential predictors will be selected based on previous literature and expert opinion.[30 31 44] Predictors will be defined by codes in the primary care records, using code lists from previous studies (via www.clinicalcodes.org). Each model will also incorporate basic patient characteristics, blood pressure level, co-morbidities and frailty, estimated from the electronic frailty index.

O. Data/ Statistical Analysis

Prognostic modelling

Aim 1 – prognostic model derivation (primary analysis)

Where feasible and appropriate, flexible parametric survival models will be derived in the CPRD GOLD for each outcome separately. Baseline survival over time will be estimated using the Royston-Parmar approach of restricted cubic splines on the cumulative log hazard scale. In contrast to Cox regression, this allows a smoothed estimate of the baseline survival function to be derived, and the time period of prediction to be varied, facilitating individualised risk prediction. For the primary analysis, a 10-year period of prediction will be considered. Further sensitivity analyses will be conducted examining a 5-year period. Where insufficient data are available, or modelling procedures become prohibitively complex (computationally), simpler approaches such as Cox regression will be considered.

Each drug class (prescribed in the 12 months prior to the index date) will be entered into the model as a binary variable to allow for multiple treatments to be considered in each model. Where appropriate, models will be constructed with all other variables entered as continuous (not categorised) variables, and potential non-linear trends will be examined. Transformations or fractional polynomials will be used if non-linear trends are detected. Where hazard ratios are not proportional, interactions of effects with time will be included. Further interaction terms, identified in an accompanying systematic review, accounting for treatment effects which differ across specific populations may be included. Where appropriate, models will be reduced and candidate predictors selected for inclusion based on p values of association. Time since cohort entry will be used as the underlying time function in each survival model.

In a further analysis, we will extend the modelling process as described above to account for competing risk of death from other causes. This will produce a model that predicts the risk of adverse events from cardiovascular treatment over time, in the real world where individuals might die from other causes and thus prevent cardiovascular treatment adverse events from occurring. We will again use a flexible parametric modelling framework to estimate the sub-distribution hazard and cause-specific cumulative incidence functions.[45]

Missing data will be handled, under a missing at random assumption, using multiple imputation (see below) followed by Rubin's rules to combine parameter estimates across studies.

Where feasible and appropriate, each model will be internally validated using the bootstrap method, to allow optimism-adjusted estimates of calibration and discrimination performance to be obtained. Adjustment for overfitting will be undertaken using a uniform shrinkage factor identified from bootstrapping, followed by re-estimation of the cumulative baseline sub-distribution hazard function (and thus baseline cumulative incidence function) to maintain overall calibration between observed and predicted risks. Given the large sample size, overfitting is likely to be small (see sample size calculation above). If bootstrapping is computationally infeasible due to the large sample size, then a smaller random sample of the population will be used or alternative internal validation methods employed such as cross validation.

Aim 2 – prognostic model validation

External model validation will be undertaken using data from CPRD Aurum. The accuracy of risk predictions from each model will be assessed with calibration plots and 5 and 10 years, calibration measures (slope, E/O statistics), measures of overall fit (using pseudo-R^2 estimates) and discrimination measures (C-statistics and D-statistics).

Aim 3 – causal inference analysis

For most of the outcomes described in this proposal, the association and magnitude of effect with treatment is not well defined in randomised controlled trials. We will therefore undertake causal inference work to examine whether modification of treatment could have an important impact on the risk of adverse events using two causal inference methods: propensity score matching and instrumental variable analysis.

For the propensity score analysis, eligible patients will be matched 1:1 at the index date using propensity scores, which indicate the likelihood a patient will be prescribed treatment on the basis of their known (pre-treatment) characteristics and other known information which might influence the decision to treat. Predictors of preventative drug prescription will be explored in a logistic regression model. Matched patients will be compared using the Cox proportional hazards models, but hazard ratios will only be adjusted by factors unbalanced at baseline which are not already included in the propensity score model. Time since cohort entry will be used as the underlying time function in each model.

For the instrumental variable analysis, GP's previous antihypertensive prescribing preferences will be used as an instrument to predict the likelihood of actual treatment. Risk of adverse events will be estimated in patients attending a GP considered a high prescriber of preventative medication and compared to those attending a GP considered a low prescriber. If the results from the propensity score matching suffer from unmeasured confounding, they will differ from those of the instrumental analysis. If this is the case, these models will be refined with additional factors to achieve better adjustment and matching. Where all approaches provide similar results, we can be more confident that the findings are accurate and reliable.[46]

A similar approach will be applied to secondary outcomes, for example, memory loss which has been hypothesised as being linked with statins[47] and is a concern of many patients in practice, but there is no evidence to support this association from trials.[48] We recognise that the evidence base may change for some outcomes over the next few years, and so the need to test the associations between treatment and certain outcomes may change.

Subgroup and sensitivity analyses

Where possible and appropriate, analyses of treatment associations will be examined in subgroups of the population. These will include age, sex, baseline blood pressure and cardiovascular risk, by drug type/dose/intensity and past medical history. Subgroup definitions will be agreed by expert opinion or taken from an accompanying systematic review of previous trials, examining the same topic.

Sensitivity analyses will be undertaken examining risk models for outcomes defined according to both hospital and primary care records. We will also explore the impact of our definition of myopathy and muscle pain, focussing on formal diagnostic codes for myopathy only. Models utilising a 5-year follow-up will be examined as a further sensitivity analysis. The primary analyses will examine the association between treatment and outcomes, assuming treatments are not altered or modified in the future. Where possible, sensitivity analyses will examine treatment entered as time varying covariates, and censor patients who have treatments removed or switched following the index date.

P. Plan for addressing confounding

Two approaches will be taken to determine whether observed associations are potentially subject to residual confounding.[46] Where all approaches provide similar results, we can be more confident that the findings are accurate and reliable. Further steps will be taken to examine the validity of treatment effect estimates using positive and negative controls: the impact of treatment on an outcome known to be affected by treatment (e.g. cardiovascular disease; positive control) and outcomes not known to be affected by treatment (negative control). Here, we will combine two common but unrelated outcomes to be examined as negative controls: bowel cancer and chronic obstructive pulmonary disease (COPD). If treatment has a significant impact on these negative controls, it suggests that there is something missing in the analysis (i.e. an unmeasurable factor confounding the treatment effect such as being generally unwell or an unhealthy lifestyle) causing an imbalance between the treatment and control groups, rather than a true treatment effect.

Q. Plans for addressing missing data

There is potential for missing data in this study, particularly with variables such as ethnicity which are recorded with varying degrees of accuracy in routine practice. Because this analysis is focused on treatment for prevention of cardiovascular disease, accurate selection of the sample population is important and therefore any patients with insufficient data available to define their blood pressure or cardiovascular risk status at baseline (index date) will be excluded. Where there is no record of blood pressure lowering, statin or antiplatelet treatment, it will be assumed the patients were not exposed to blood pressure lowering, statin or antiplatelet treatment.

Patient eligibility includes only using ‘acceptable patients’ in the analysis, and therefore there is no need to impute age and sex. Where there is no record of smoking history or alcohol consumption, patients will be assumed to be non-smokers and non-drinkers. Likewise, those with no record of co-morbidities will be assumed to have no history of these conditions. All other covariates (including BMI, ethnicity and IMD) used in the prognostic or casual inference modelling will be imputed using multiple imputation. BMI and will be treated as a continuous variable.

All analyses of treatment associations with outcomes will be conducted by intention-to-treat, and patients moving practice after the index date (and therefore being lost to follow-up) will be censored at the point at which they are no longer active in the database.

R. Patient or user group involvement (if applicable)

This application has been discussed with patients (aged 67-86 years, taking 5-11 meds), a carer who also takes multiple meds takes 5 meds and the AgeUK Bakewell day centre (group discussion with 18 frail elderly persons).

Key issues raised

- Individuals vary widely on the amount of information they wish to know about their medications, and this is often influenced by the doctor-patient relationship.
- Patients sometimes rely on unclear or unreliable information about the potential harms of their treatments from medication packets, the internet or friends.
- Some patients are concerned about suffering events which will cause them to go to hospital, but most focus on the 'here-and-now'; chronic issues which affect their quality of life (e.g. pain limiting mobility).
- Some do not trust their GPs understanding of medications and prefer to see a specialist pharmacist with more time to consider their needs. Others who see the same GP regularly are happy to 'do as they are told,' but assume the GP fully understands the benefits and harms of the treatments they take.
- Patients are happy to be involved in research, but location and access is very important, and a multifaceted approach to engagement is required. Some are more comfortable with technology than others.
- Older individuals said they were happier having (potentially sensitive) PPI discussions with someone they knew and trusted.

Project areas directly influenced by PPI

Overarching aim: Some individuals wish to know about the benefits and harms of medications whilst others expect the GP will fully understand the risks. A clinical decision support tool is needed to improve the understanding of both patients and their GPs.

Model outcomes: Patient's opinions vary in the outcomes that concern them the most and so this proposal focusses on both serious acute events and also chronic problems which can affect daily life. Advice will be sought as to how these outcomes should be defined in the final calculators.

PPI engagement: A variety of methods of PPI engagement are required to maximise access for patients. Engaging with relatives and familiar members of local churches and community groups will be important.

S. Plans for disseminating and communicating study results, including the presence or absence of any restrictions on the extent and timing of publication

All findings from the proposed research project will be published in peer-reviewed scientific journals. Findings will be presented at national and international conferences in Primary Care (e.g. Society for Academic Primary Care [SAPC], North American Primary Care Research Group), Hypertension (British and Irish Hypertension Society [BIHS], European Society for Hypertension) and cardiovascular disease (European Society of Cardiology).

An article discussing the issues raised by the research will be written for the online newspaper 'The Conversation', and this will also be posted on the Nuffield Department of Primary Care Health Sciences website as an online blog. Where appropriate, results of the research will be press-released in combination with their publication in scientific journals. Social media (twitter) will be used to draw further attention to the work.

The results of this work will be used to develop a calculator which estimates an individual's risk of suffering harm from preventative treatments for cardiovascular disease. The risk calculator will be made freely available online for use by patients and practitioners.

Conflict of interest statement: The authors declare no conflicts of interest

T. Limitations of the study design, data sources, and analytic methods

This study focuses on potential harms from adverse events associated with preventative treatments for cardiovascular disease. Such outcomes may be less well recorded in routine electronic health records. For example, initial scoping work suggests the rate of falls documented in the CPRD is between 0.06-0.34% per 1000 person-months. These incidence rates may be less than those reported in smaller previous studies (0.15-6.40% per 1000 person-months) using different definitions, captured via detailed questionnaires and follow-up.[49-52] Incidence rates are likely to be higher when linked data from HES and ONS are available.[53] Missing data on certain outcomes may reduce the accuracy of the prognostic models, but should not bias the results provided the data are missing at random and not affected by whether patients are prescribed treatment. To minimise the risk of reporting bias, outcomes in the primary analysis will be defined according to hospital admissions documented in the HES or ONS.

The causal inference work will use an observational cohort design, and as such, there is an inherent selection bias of patients in both exposure and control groups. The impact of this bias will be limited by propensity score methods used to control for confounding, but this approach assumes that all confounding factors are measured and accounted for within the analysis. In the present study, we will examine the validity of this assumption by 1) using an instrumental variable (GP prescriber preference) and examining the association with the outcomes of interest and 2) by studying the impact of treatment on positive and negative controls: hospitalisation and/or death from cardiovascular disease and bowel cancer/COPD. We will conclude the assumption has been met if high GP prescribers have a similar association with outcomes and the treatment itself and treatment is shown to be associated with the positive control, but not the negative control.

Strategy for dealing with potential errors resulting from multiple testing

This study will examine multiple outcomes across 3 exposure variables. To avoid potential errors arising from multiple testing, a primary outcome has been clearly defined for each exposure variable. Secondary outcomes are also pre-specified. The primary outcome will be given priority in the final analysis write-up and any related reports and presentations.

Primary and secondary outcomes will be defined using data from Basic inpatient HES and ONS. Sensitivity analyses will explore analyses defining these outcomes using the above sources and read-coded primary care data. The accuracy of such outcome data has been examined previously in patients with acute myocardial infarction and whilst recording of risk factor and co-morbid information was consistent across primary care, hospital admissions and disease registry records, the crude incidence of acute myocardial infarction was underestimated by up to 50% if only one data source was used, compared with using all three sources.[53] The use of linked CPRD data will ensure outcome data are ascertained accurately.

U. References

1. Lozano R, Naghavi M, Foreman K, et al. Global and regional mortality from 235 causes of death for 20 age groups in 1990 and 2010: a systematic analysis for the Global Burden of Disease Study 2010. *Lancet* 2012;380(9859):2095-128. doi: 10.1016/s0140-6736(12)61728-0 [published Online First: 2012/12/19]

2. NatCen Social Research. Health Survey for England 2014. http://digital.nhs.uk/catalogue/PUB19297, 2015.

3. Chou R, Dana T, Blazina I, et al. Statins for Prevention of Cardiovascular Disease in Adults: Evidence Report and Systematic Review for the US Preventive Services Task Force. *Jama* 2016;316(19):2008-24. doi: 10.1001/jama.2015.15629 [published Online First: 2016/11/14]

4. Ettehad D, Emdin CA, Kiran A, et al. Blood pressure lowering for prevention of cardiovascular disease and death: a systematic review and meta-analysis. *Lancet* 2016;387(10022):957-67. doi: 10.1016/s0140-6736(15)01225-8 [published Online First: 2016/01/03]

5. Collaborative meta-analysis of randomised trials of antiplatelet therapy for prevention of death, myocardial infarction, and stroke in high risk patients. *BMJ (Clinical research ed)* 2002;324(7329):71-86. [published Online First: 2002/01/12]

6. Sheppard JP, Stevens S, Stevens R, et al. Benefits and Harms of Antihypertensive Treatment in Low-Risk Patients With Mild Hypertension. *JAMA internal medicine* 2018;178(12):1626-34. doi: 10.1001/jamainternmed.2018.4684 [published Online First: 2018/11/02]

7. Wright JT, Jr., Williamson JD, Whelton PK, et al. A Randomized Trial of Intensive versus Standard Blood-Pressure Control. *The New England journal of medicine* 2015;373(22):2103-16. doi: 10.1056/NEJMoa1511939 [published Online First: 2015/11/10]

8. Callisaya ML, Sharman JE, Close J, et al. Greater daily defined dose of antihypertensive medication increases the risk of falls in older people--a population-based study. *J Am Geriatr Soc* 2014;62(8):1527-33. doi: 10.1111/jgs.12925 [published Online First: 2014/06/18]

9. Tinetti ME, Han L, Lee DS, et al. Antihypertensive medications and serious fall injuries in a nationally representative sample of older adults. *JAMA internal medicine* 2014;174(4):588-95. doi: 10.1001/jamainternmed.2013.14764 [published Online First: 2014/02/26]

10. Sinnott C, Hugh SM, Boyce MB, et al. What to give the patient who has everything? A qualitative study of prescribing for multimorbidity in primary care. *The British journal of general practice : the journal of the Royal College of General Practitioners* 2015;65(632):e184-91. doi: 10.3399/bjgp15X684001 [published Online First: 2015/03/04]

11. Ioannidis JP, Lau J. Completeness of safety reporting in randomized trials: an evaluation of 7 medical areas. *Jama* 2001;285(4):437-43. [published Online First: 2001/03/10]

12. Stevens PE, Lamb EJ, Levin A. Integrating guidelines, CKD, multimorbidity, and older adults. *American journal of kidney diseases : the official journal of the National Kidney Foundation* 2015;65(3):494-501. doi: 10.1053/j.ajkd.2014.09.024 [published Online First: 2014/12/09]

13. Gribbin J, Hubbard R, Gladman JR, et al. Risk of falls associated with antihypertensive medication: population-based case-control study. *Age and ageing* 2010;39(5):592-7. doi: 10.1093/ageing/afq092 [published Online First: 2010/07/24]

14. Butt DA, Mamdani M, Austin PC, et al. The risk of falls on initiation of antihypertensive drugs in the elderly. *Osteoporosis international : a journal established as result of cooperation between the European Foundation for Osteoporosis and the National Osteoporosis Foundation of the USA* 2013;24(10):2649-57. doi: 10.1007/s00198-013-2369-7 [published Online First: 2013/04/25]

15. Mansfield KE, Nitsch D, Smeeth L, et al. Prescription of renin–angiotensin system blockers and risk of acute kidney injury: a population-based cohort study. *BMJ open* 2016;6(12) doi: 10.1136/bmjopen-2016-012690

16. Hippisley-Cox J, Coupland C. Unintended effects of statins in men and women in England and Wales: population based cohort study using the QResearch database. *BMJ (Clinical research ed)* 2010;340:c2197. doi: 10.1136/bmj.c2197 [published Online First: 2010/05/22]

17. Macedo AF, Taylor FC, Casas JP, et al. Unintended effects of statins from observational studies in the general population: systematic review and meta-analysis. *BMC medicine* 2014;12:51. doi: 10.1186/1741-7015-12-51 [published Online First: 2014/03/25]

18. Garcia Rodriguez LA, Martin-Perez M, Hennekens CH, et al. Bleeding Risk with Long-Term Low-Dose Aspirin: A Systematic Review of Observational Studies. *PLoS ONE* 2016;11(8):e0160046. doi: 10.1371/journal.pone.0160046 [published Online First: 2016/08/05]

19. Starfield B. New paradigms for quality in primary care. *The British journal of general practice : the journal of the Royal College of General Practitioners* 2001;51(465):303-9.

20. Lip GY, Nieuwlaat R, Pisters R, et al. Refining clinical risk stratification for predicting stroke and thromboembolism in atrial fibrillation using a novel risk factor-based approach: the euro heart survey on atrial fibrillation. *Chest* 2010;137(2):263-72. doi: 10.1378/chest.09-1584 [published Online First: 2009/09/19]

21. Pisters R, Lane DA, Nieuwlaat R, et al. A novel user-friendly score (HAS-BLED) to assess 1-year risk of major bleeding in patients with atrial fibrillation: the Euro Heart Survey. *Chest* 2010;138(5):1093-100. doi: 10.1378/chest.10-0134 [published Online First: 2010/03/20]

22. Damen JA, Hooft L, Schuit E, et al. Prediction models for cardiovascular disease risk in the general population: systematic review. *BMJ (Clinical research ed)* 2016;353:i2416. doi: 10.1136/bmj.i2416 [published Online First: 2016/05/18]

23. Anderson KM, Odell PM, Wilson PW, et al. Cardiovascular disease risk profiles. *American heart journal* 1991;121(1 Pt 2):293-8. [published Online First: 1991/01/01]

24. D'Agostino RB, Sr., Vasan RS, Pencina MJ, et al. General cardiovascular risk profile for use in primary care: the Framingham Heart Study. *Circulation* 2008;117(6):743-53. doi: 10.1161/circulationaha.107.699579 [published Online First: 2008/01/24]

25. Woodward M, Brindle P, Tunstall-Pedoe H. Adding social deprivation and family history to cardiovascular risk assessment: the ASSIGN score from the Scottish Heart Health Extended Cohort (SHHEC). *Heart (British Cardiac Society)* 2007;93(2):172-6. doi: 10.1136/hrt.2006.108167 [published Online First: 2006/11/09]

26. Hippisley-Cox J, Coupland C, Vinogradova Y, et al. Predicting cardiovascular risk in England and Wales: prospective derivation and validation of QRISK2. *BMJ (Clinical research ed)* 2008;336(7659):1475-82. doi: 10.1136/bmj.39609.449676.25 [published Online First: 2008/06/25]

27. Joint British Societies. Joint British Societies' consensus recommendations for the prevention of cardiovascular disease (JBS3). *Heart (British Cardiac Society)* 2014;100 Suppl 2:ii1-ii67. doi: 10.1136/heartjnl-2014-305693 [published Online First: 2014/03/29]

28. Hippisley-Cox J, Coupland C, Brindle P. Development and validation of QRISK3 risk prediction algorithms to estimate future risk of cardiovascular disease: prospective cohort study. *BMJ (Clinical research ed)* 2017;357:j2099. doi: 10.1136/bmj.j2099 [published Online First: 2017/05/26]

29. Birnie K, Verheyden V, Pagano D, et al. Predictive models for kidney disease: improving global outcomes (KDIGO) defined acute kidney injury in UK cardiac surgery. *Critical care (London, England)* 2014;18(6):606. doi: 10.1186/s13054-014-0606-x [published Online First: 2015/02/13]

30. Englberger L, Suri RM, Li Z, et al. Validation of clinical scores predicting severe acute kidney injury after cardiac surgery. *American journal of kidney diseases : the official journal of the National Kidney Foundation* 2010;56(4):623-31. doi: 10.1053/j.ajkd.2010.04.017 [published Online First: 2010/07/16]

31. Hodgson LE, Dimitrov BD, Roderick PJ, et al. Predicting AKI in emergency admissions: an external validation study of the acute kidney injury prediction score (APS). *BMJ open* 2017;7(3):e013511. doi: 10.1136/bmjopen-2016-013511 [published Online First: 2017/03/10]

32. Wang YN, Cheng H, Yue T, et al. Derivation and validation of a prediction score for acute kidney injury in patients hospitalized with acute heart failure in a Chinese cohort. *Nephrology (Carlton, Vic)* 2013;18(7):489-96. doi: 10.1111/nep.12092 [published Online First: 2013/04/24]

33. Hippisley-Cox J, Coupland C. Derivation and validation of updated QFracture algorithm to predict risk of osteoporotic fracture in primary care in the United Kingdom: prospective open cohort study. *BMJ (Clinical research ed)* 2012;344:e3427. doi: 10.1136/bmj.e3427 [published Online First: 2012/05/24]

34. Kanis JA, Johnell O, Oden A, et al. FRAX™ and the assessment of fracture probability in men and women from the UK. *Osteoporosis international : a journal established as result of cooperation between the European Foundation for Osteoporosis and the National Osteoporosis Foundation of the USA* 2008;19(4):385-97. doi: 10.1007/s00198-007-0543-5

35. Jennings LA, Reuben DB, Kim SB, et al. Targeting a high-risk group for fall prevention: strategies for health plans. *American Journal of Managed Care* 2015;21(9):e519-26.

36. Stalenhoef PA, Diederiks JP, Knottnerus JA, et al. A risk model for the prediction of recurrent falls in community-dwelling elderly: a prospective cohort study. *Journal of Clinical Epidemiology* 2002;55(11):1088-94.

37. Woo J, Leung J, Wong S, et al. Development of a simple scoring tool in the primary care setting for prediction of recurrent falls in men and women aged 65 years and over living in the community. *Journal of Clinical Nursing* 2009;18(7):1038-48.

38. Basu S, Sussman JB, Rigdon J, et al. Benefit and harm of intensive blood pressure treatment: Derivation and validation of risk models using data from the SPRINT and ACCORD trials. *PLoS medicine* 2017;14(10):e1002410. doi: 10.1371/journal.pmed.1002410 [published Online First: 2017/10/19]

39. Patel KK, Arnold SV, Chan PS, et al. Personalizing the Intensity of Blood Pressure Control: Modeling the Heterogeneity of Risks and Benefits From SPRINT (Systolic Blood Pressure Intervention Trial). *Circulation Cardiovascular quality and outcomes* 2017;10(4) doi: 10.1161/circoutcomes.117.003624 [published Online First: 2017/04/05]

40. Riley RD, Snell KIE, Ensor J, et al. Minimum sample size for developing a multivariable prediction model: Part I - Continuous outcomes. *Stat Med* 2018 doi: 10.1002/sim.7993 [published Online First: 2018/10/23]

41. van Asch CJ, Luitse MJ, Rinkel GJ, et al. Incidence, case fatality, and functional outcome of intracerebral haemorrhage over time, according to age, sex, and ethnic origin: a systematic review and meta-analysis. *The Lancet Neurology* 2010;9(2):167-76. doi: 10.1016/s1474-4422(09)70340-0 [published Online First: 2010/01/09]

42. Stevens SL, McManus RJ, Stevens RJ. The utility of long-term blood pressure variability for cardiovascular risk prediction in primary care. *J Hypertension* 2018 doi: 10.1097/hjh.0000000000001923 [published Online First: 2018/09/21]

43. Vergouwe Y, Steyerberg EW, Eijkemans MJ, et al. Substantial effective sample sizes were required for external validation studies of predictive logistic regression models. *J Clin Epidemiol* 2005;58(5):475-83. doi: 10.1016/j.jclinepi.2004.06.017 [published Online First: 2005/04/23]

44. National Guideline Centre. National Institute for Health and Care Excellence: Clinical Guidelines. Falls in older people: assessing risk and prevention [Clinical Guideline 161]. London: National Institute for Health and Care Excellence (UK)
Copyright (c) National Institute for Health and Care Excellence, 2016. 2013.

45. Lambert PC, Wilkes SR, Crowther MJ. Flexible parametric modelling of the cause-specific cumulative incidence function. *Stat Med* 2017;36(9):1429-46. doi: 10.1002/sim.7208 [published Online First: 2016/12/23]

46. Taylor GMJ, Taylor AE, Thomas KH, et al. The effectiveness of varenicline versus nicotine replacement therapy on long-term smoking cessation in primary care: a prospective cohort study of electronic medical records. *International journal of epidemiology* 2017;46(6):1948-57. doi: 10.1093/ije/dyx109 [published Online First: 2017/10/19]

47. Strom BL, Schinnar R, Karlawish J, et al. Statin Therapy and Risk of Acute Memory Impairment. *JAMA internal medicine* 2015;175(8):1399-405. doi: 10.1001/jamainternmed.2015.2092 [published Online First: 2015/06/09]

48. Collins R, Reith C, Emberson J, et al. Interpretation of the evidence for the efficacy and safety of statin therapy. *Lancet* 2016;388(10059):2532-61. doi: 10.1016/s0140-6736(16)31357-5 [published Online First: 2016/09/13]

49. Ali T, Khan I, Simpson W, et al. Incidence and outcomes in acute kidney injury: a comprehensive population-based study. *Journal of the American Society of Nephrology : JASN* 2007;18(4):1292-8. doi: 10.1681/asn.2006070756 [published Online First: 2007/02/23]

50. Barton AL, Mallard AS, Parry RG. One Year's Observational Study of Acute Kidney Injury Incidence in Primary Care; Frequency of Follow-Up Serum Creatinine and Mortality Risk. *Nephron* 2015;130(3):175-81.

51. Stalenhoef PA, Diederiks JP, de Witte LP, et al. Impact of gait problems and falls on functioning in independent living persons of 55 years and over: a community survey. *Patient education and counseling* 1999;36(1):23-31. [published Online First: 1999/02/26]

52. Tinetti ME, Speechley M, Ginter SF. Risk factors for falls among elderly persons living in the community. *The New England journal of medicine* 1988;319(26):1701-7. doi: 10.1056/nejm198812293192604 [published Online First: 1988/12/29]

53. Herrett E, Shah AD, Boggon R, et al. Completeness and diagnostic validity of recording acute myocardial infarction events in primary care, hospital care, disease registry, and national mortality records: cohort study. *BMJ (Clinical research ed)* 2013;346 doi: 10.1136/bmj.f2350

List of Appendices

Appendix 1. Code lists for the primary outcomes

Amendments

Amendment 1 – 19/05/2019 (approved)

We clarify that aims 1 and 2 (prognostic model derivation and validation) of the study should include the whole population independently of whether patients are new or old users of preventative treatment; the causal inference analyses will focus on new users only:

L. Definition of the study population

Patients will be excluded if they meet the following criteria:

Previous prescription of the exposure variable (antihypertensive, statin or antiplatelet; for causal inference analyses only)

Amendment 2 – 28/06/2021 (approved 05/07/2021)

We have made the following revisions aimed at simplifying the analysis plan in light of the very large sample size and computational time required to conduct each analysis:

C. Technical Summary / E. Objectives, Specific Aims and Rationale

Aims:

3) Use causal inference methods (multivariable regression, propensity score adjustment/matching, instrumental variable analysis) to examine whether modification of treatment could have an important impact on the risk of adverse events.

L. Definition of the study population

Study entry criteria – Patients will enter the cohort on the index date, defined as:

**Exposure**	Antihypertensive therapy	Statin therapy	Antiplatelet therapy
**Index date definition**	12 months after the first systolic blood pressure reading ≥130 mm Hg	12 months after the first coded or calculated CVD risk of ≥5% (equivalent to age ≥50 years for men and ≥60 years for women)	3 months after first CVD event

N. Exposures, Outcomes and Covariates

Positive controls are outcomes know to be affected by treatment (e.g. cardiovascular disease, death)

For causal inference analyses (only), sensitivity analyses will explore outcomes defined more broadly using primary care codes as well.

O. Data/ Statistical Analysis

We no longer plan to conduct an internal validation of the prediction models. The following text from the protocol is therefore redundant:

Where feasible and appropriate, each model will be internally validated using the bootstrap method, to allow optimism-adjusted estimates of calibration and discrimination performance to be obtained. Adjustment for overfitting will be undertaken using a uniform shrinkage factor identified from bootstrapping, followed by re-estimation of the cumulative baseline sub-distribution hazard function (and thus baseline cumulative incidence function) to maintain overall calibration between observed and predicted risks. Given the large sample size, overfitting is likely to be small (see sample size calculation above). If bootstrapping is computationally infeasible due to the large sample size, then a smaller random sample of the population will be used or alternative internal validation methods employed such as cross validation.

Aim 3 – causal inference analysis

For most of the outcomes described in this proposal, the association and magnitude of effect with treatment is not well defined in randomised controlled trials. We will therefore undertake causal inference work to examine whether modification of treatment could have an important impact on the risk of adverse events using two causal inference methods: multivariable adjustment, propensity score adjustment/matching and instrumental variable analysis.

For the analysis using multivariable adjustment, Cox proportional hazards models will be used to compare adverse event rates in each experimental group. Estimates will be adjusted for pre-specified variables thought to confound the relationship between treatment and adverse events.

For the analysis using propensity scores, we will conduct separate analyses adjusting for propensity score and matching via propensity score.

Subgroup and sensitivity analyses

Where possible and appropriate, analyses of treatment associations will be examined in subgroups of the population. These will include age, sex, baseline blood pressure and cardiovascular risk, by drug type/dose/intensity and past medical history. Subgroup definitions will be agreed by expert opinion or taken from an accompanying systematic review of previous trials, examining the same topic. For computational reasons (whereby only one imputation model is required for all subgroup analyses of each outcome), these analyses will only be undertaken using propensity score adjustment.

P. Plan for addressing confounding

Two approaches will be taken to determine whether observed associations are potentially subject to residual confounding.[46] Where all approaches provide similar results, we can be more confident that the findings are accurate and reliable. Further steps will be taken to examine the validity of treatment effect estimates using positive and negative controls: the impact of treatment on an outcome known to be affected by treatment (e.g. cardiovascular disease and death; positive controls)

Amendment 3 – 25.11.2021 (approved 03/12/2021)

We have added exploratory analyses using supervised machine learning approaches to address missing data and deal with confounding by indication in the causal inference analyses.

O. Data/ Statistical Analysis

Aim 3 – causal inference analysis

For most of the outcomes described in this proposal, the association and magnitude of effect with treatment is not well defined in randomised controlled trials. We will therefore undertake causal inference work to examine whether modification of treatment could have an important impact on the risk of adverse events using two causal inference methods: propensity score matching and instrumental variable analysis.

For the propensity score analysis, eligible patients will be matched 1:1 at the index date using propensity scores, which indicate the likelihood a patient will be prescribed treatment on the basis of their known (pre-treatment) characteristics and other known information which might influence the decision to treat. Predictors of preventative drug prescription will be explored in a logistic regression model. Matched patients will be compared using the Cox proportional hazards models, but hazard ratios will only be adjusted by factors unbalanced at baseline which are not already included in the propensity score model. Time since cohort entry will be used as the underlying time function in each model.

For the instrumental variable analysis, GP's previous antihypertensive prescribing preferences will be used as an instrument to predict the likelihood of actual treatment. Risk of adverse events will be estimated in patients attending a GP considered a high prescriber of preventative medication and compared to those attending a GP considered a low prescriber. If the results from the propensity score matching suffer from unmeasured confounding, they will differ from those of the instrumental analysis. If this is the case, these models will be refined with additional factors to achieve better adjustment and matching. Where all approaches provide similar results, we can be more confident that the findings are accurate and reliable.[46]

Generative Adversarial Networks (GAN) will also be used to impute missing counterfactual data (unobserved effects of untaken treatments) on an individual level. GAN models are a class of machine learning frameworks that involves the simultaneous training of a pair of deep neural networks in competition with each other. The idea is that the first network, the generator, generates synthetic data samples that are similar to the original samples, while the second network, the discriminator, evaluates the authenticity of the

generated samples [1]. Data similarity and fidelity are usually evaluated using well-known metrics such as Maximum Mean Discrepancies [2] and Jensen-Shannon Divergence [3]. While GAN's initial uses were for imaging applications, its generative capabilities make it naturally suitable for generating missing values that can be used to impute the original samples.

In this work, we build on the intuition that treatment effects estimation and causal inference are a missing data problem, where counterfactuals (unobserved effects of untaken treatments) are missing [4]. Specifically, we plan to use GAN to impute missing counterfactual data, for the samples available in CPRD. By modifying the generator's goal to accurately impute missing data, and the discriminator's goal to distinguish between observed and imputed counterfactual values for the same patient record. The model can generate the missing data conditional on the observed values and assigned treatment for each patient as seen in [5, 6]. While useful, the work of [5, 6] are not compatible for time-series data nor for estimating multiple treatment effects, which limits its use in real-life treatment applications. Therefore, we plan to extend such previous works to make them compatible for multiple treatment options and for time-series data to allow for long-term treatment (antihypertensives, statins, antiplatelets) estimation effects for multi-morbid populations as initially proposed in this project.

The proposed GAN approach does not generate new patient samples, but rather generates counterfactual (missing component) conditioned on observed factual data, and outputs a complete record with both factual and counterfactual outcomes to allow for treatment effect estimation for each patient. This approach is naturally immune to re-identification attacks since no new samples are generated, but we will run privacy checks and measures within the data to verify that the model is not copying potential outcomes from patients in the training sample. Example privacy measures that we plan to apply include empirical evaluations such as mathematical privacy definitions such as identifiability [7] that quantifies the probability of re-identification to ensure that no combination of attributes could reveal the identity of a patient. Other privacy evaluation methods include testing that the model is robust against membership inference attacks, where an attacker attempts to determine if a specific patient was used in the training set of the model [8]. Lastly, we will experiment with strict theoretical guarantees such as differential privacy which allow for the model to learn almost nothing about an individual while learning useful information about a population [9].

References

[1] Ian Goodfellow, Jean Pouget-Abadie, Mehdi Mirza, Bing Xu, David Warde-Farley, Sherjil Ozair, Aaron Courville, and Yoshua Bengio. Generative adversarial nets. *Advances in neural information processing systems*, 27:2672–2680, 2014.

[2] Arthur Gretton, Karsten M Borgwardt, Malte J Rasch, Bernhard Sch¨olkopf, and Alexander Smola. A kernel two-sample test. *The Journal of Machine Learning Research*, 13(1):723–773, 2012.

[3] AP Majtey, PW Lamberti, and DP Prato. Jensen-shannon divergence as a measure of distinguishability between mixed quantum states. *Physical Review A*, 72(5):052310, 2005.

[4] Peng Ding and Fan Li. Causal inference: A missing data perspective. *Statistical Science*, 33(2):214–237, 2018.

[5] Jinsung Yoon, James Jordon, and Mihaela Schaar. Gain: Missing data imputation using generative adversarial nets. In *International Conference on Machine Learning*, pages 5689–5698. PMLR, 2018.

[6] Jinsung Yoon, James Jordon, and Mihaela van der Schaar. Ganite: Estimation of individualized treatment effects using generative adversarial nets. In *International Conference on Learning Representations*, 2018.

[7] Jinsung Yoon, Lydia N Drumright, and Mihaela Van Der Schaar. Anonymization through data synthesis using generative adversarial networks (ads-gan). *IEEE journal of biomedical and health informatics*, 24(8):2378–2388, 2020.

[8] Reza Shokri, Marco Stronati, Congzheng Song, and Vitaly Shmatikov. Membership inference attacks against machine learning models. In *2017 IEEE Symposium on Security and Privacy (SP)*, pages 3–18. IEEE, 2017.

[9] Cynthia Dwork, Aaron Roth, et al. The algorithmic foundations of differential privacy. *Found. Trends Theor. Comput. Sci.*, 9(3-4):211–407, 2014.
